# Epigenome-wide DNA methylation profiling in comparison between pathological and physiological hypertrophy of human cardiomyocytes

**DOI:** 10.3389/fgene.2023.1264382

**Published:** 2023-09-27

**Authors:** Hangchuan Shi, Si Chen, Fanju W. Meng, Deborah J. Ossip, Chen Yan, Dongmei Li

**Affiliations:** ^1^ Department of Clinical and Translational Research, University of Rochester Medical Center, Rochester, NY, United States; ^2^ Department of Public Health Sciences, University of Rochester Medical Center, Rochester, NY, United States; ^3^ Aab Cardiovascular Research Institute, University of Rochester, School of Medicine and Dentistry, Rochester, NY, United States; ^4^ Department of Biomedical Genetics, University of Rochester Medical Center, Rochester, NY, United States

**Keywords:** cardiac hypertrophy, DNA methylation, PI3K-Akt, Hippo, signaling, *CDK6*, *RPTOR*

## Abstract

**Background:** Physiological and pathological stimuli result in distinct forms of cardiac hypertrophy, but the molecular regulation comparing the two, especially at the DNA methylation level, is not well understood.

**Methods:** We conducted an *in vitro* study using human cardiomyocytes exposed to angiotensin II (AngII) and insulin-like growth factor 1 (IGF-1) to mimic pathologically and physiologically hypertrophic heart models, respectively. Whole genome DNA methylation patterns were profiled by the Infinium human MethylationEPIC platform with >850 K DNA methylation loci. Two external datasets were used for comparisons and qRT-PCR was performed for examining expression of associated genes of those identified DNA methylation loci.

**Results:** We detected 194 loci that are significantly differentially methylated after AngII treatment, and 206 significant loci after IGF-1 treatment. Mapping the significant loci to genes, we identified 158 genes corresponding to AngII treatment and 175 genes to IGF-1 treatment. Using the gene-set enrichment analysis, the PI3K-Akt signaling pathway was identified to be significantly enriched for both AngII and IGF-1 treatment. The Hippo signaling pathway was enriched after IGF-1 treatment, but not for AngII treatment. *CDK6* and *RPTOR* are components of the PI3K-Akt pathway but have different DNA methylation patterns in response to AngII and IGF-1. qRT-PCR confirmed the different gene expressions of *CDK6* and *PRTOR*.

**Conclusion:** Our study is pioneering in profiling epigenome DNA methylation changes in adult human cardiomyocytes under distinct stress conditions: pathological (AngII) and physiological (IGF-1). The identified DNA methylation loci, genes, and pathways might have the potential to distinguish between pathological and physiological cardiac hypertrophy.

## Introduction

Physiological and pathological stimuli result in distinct anatomic forms of cardiac hypertrophy through different molecular mechanisms (Walsh, 2006). Physiological cardiac hypertrophy presents as an increase in cardiac muscle cell diameter, which is observed in the hearts of trained athletes. The physiological growth of heart is a result of the adaptation to the increased workload, leading to increased vascularization of the myocardium and generation of more forceful ejection (Hudlicka and Brown, 1996; Richey and Brown, 1998). In contrast, pathological hypertrophy occurs in cardiovascular disease (CVD) patients (e.g., hypertension, valvular heart disease) in whom the cardiac muscle cell enlargement is coupled with cell senescence, cell death, fibrotic remodeling, and cardiac dysfunction (Shimizu and Minamino, 2016). Although some molecular distinctions have been made between physiological and pathological cardiac hypertrophy, most results were derived from studies focusing on mechanisms of pathological cardiac hypertrophy (Walsh, 2006). The comparison of molecular regulation between physiological and pathological cardiac hypertrophy is less understood.

The molecular mechanisms distinctions in adaptation to pathological and physiological cardiac hypertrophy involve the regulation and interaction among DNA, RNA, and protein. Most studies focused on the level of protein-protein interaction and gene expression, whereas the role of epigenetic modification (e.g., DNA methylation and histone modification) is less known. Recent investigations, however, have begun to shed light on the potential of DNA methylation to mediate both the physiological cardiac remodeling process induced by physical activity (Shi et al., 2021) and the pathological hypertrophy that can lead to heart failure (Liu and Tang, 2019). Although previous studies profiled whole genome-wide DNA methylation patterns by using human pathologically hypertrophic heart tissue (Haas et al., 2013; Jo et al., 2016; Ortega et al., 2018; Pepin et al., 2019), some limitations are noted. First, human heart tissue consists of a mixed type of cells not limited to cardiomyocytes, endothelial cells, inflammatory cells, and fibroblasts. Thus, the profiling of heart tissue cannot provide loci exclusively related to cardiomyocytes. While there are methods available to attempt the correction or deconvolution of bulk methylation samples by considering cell type proportions in the heart (Titus et al., 2017), the precise cellular composition of the heart remains a debated and uncertain topic (Zhou and Pu, 2016). Second, although the methylation loci which are associated with heart failure can be identified, studies did not identify loci that are influenced by physical activity, which may play important roles in preventing heart failure. In addition, it is difficult to obtain physiologically hypertrophic heart tissue as a control group due to ethical issues. Lastly, most previous studies utilized old platforms such as HumanMethylation 450K, failing to capture the advancements introduced by novel platforms that have significantly expanded the coverage of CpG sites.

To overcome the above limitations, we conducted an *in vitro* study with only 1 cell type–cardiomyocyte. We established both pathologically and physiologically hypertrophic heart models by exposing adult human cardiomyocytes to angiotensin II (AngII) and insulin-like growth factor 1 (IGF-1), respectively. AngII is released in the human body following hemodynamic overload and stimulates a cardiac hypertrophic response. Chronic unbalanced AngII will result in an uncompensated cardiac hypertrophic response, which ultimately leads to heart failure (Watkins et al., 2012). In contrast, IGF-1 was found to play a role in the induction of physiological heart growth (Weeks and McMullen, 2011). Studies showed that higher levels of IGF-1 were found in athletes compared with healthy sedentary people and was related to left ventricle end-diastolic dimension index (Neri Serneri et al., 2001). Our study was among the first to profile the whole-genome level of DNA methylation changes in the human cardiomyocytes in response to a pure pathological stimulus (i.e., AngII) and a pure physiological stimulus (i.e., IGF-1).

## Materials and methods

### Biomaterial processing

AC16 human cardiomyocyte cell line, a proliferating cell line derived from adult human ventricular cardiomyocyte with SV40 transformed (Davidson et al., 2005), was purchased from Sigma and cultured in DMEM/F-12 (Thermo Fisher Scientific) + 12.5% (v/v) FBS +1% (v/v) P/S. AC16 cells were serum starved for 24 h before treatment. For treatment, AC16 were treated with Ang II 100 nM (6 samples), IGF-1 100 ng/mL (6 samples) or vehicle treatment (i.e., control group; 4 samples) for both 12h and 24 h (further details regarding the chosen time points are provided in the Supplementary Material S1).

Genomic DNA was extracted using the Quick-DNA Isolation Kit (Zymo). For each assay, 1 μg of DNA was used for whole genome-wide DNA methylation profiling performed on the Infinium Human MehtylationEPIC BeadChip (Illumina) following the manufacturer’s recommendations. Briefly, the genomic DNA was bisulfite converted using the EZ DNA Methylation Kit (Zymo Research Corp., Irvine, CA, United States) before amplification. The samples were then whole-genome-amplified, followed incubation, fragmentation, precipitation, and resuspension. The resuspended samples were hybridized onto the BeadChip, and underwent wash, extension, and staining. After imaging, “IDAT” files were generated as the methylation array raw data. The Infinium MehtylationEPIC BeadChip array contains >850K of highly informative DNA methylation loci covering 99% of RefSeq genes, and 95% of all known CpG islands, along with enhancer sits and other content categories. Compared to its predecessor (i.e., Mehtylation450K BeadChip), MehtylationEPIC platform contains over 90% of the CpGs on the Illumina Methylatio450 plus an additional 350,000 CpGs in enhancer regions.

RNA was extracted from AC16 cells using RNeasy kit (Qiagen) according to the manufacturer’s instructions. cDNA was synthesized with the iScriptDNA synthesis kit (Bio-Rad). qPCR amplification was performed by using IQ SYBR Green Supermix (Bio-Rad). Each reaction was performed in triplicate. Primers for qPCR were as follows.

*GAPDH*

(F) TTG​ACT​CCG​ACC​TTC​ACC​TTC​C (R) CGC​TCT​CTG​CTC​CTC​CTG​TTC

*CDK6*

(F) TCA​CGA​ACA​GAC​AGA​GAA​ACC (R) CTC​CAG​GCT​CTG​GAA​CTT​TAT​C

*RPTOR*

(F) CTT​CGT​TCT​GTG​AGC​TCC​TAT​G (R) CAC​CAG​ATT​CCT​CTG​TCA​AAC​T

Primers for *COL4A6*, *CREB3L2*, *FGF12*, *GHR*, and *NRAS*, *FN1* can be found in Supplementary Material S1.

### DNA methylation profiling

The resulting raw DNA methylation data (IDAT files) were used to quantify the proportion of methylation at each DNA methylation locus on a probe-wise basis using the minfi package (1.36.0) in R3.2.2.17 (Aryee et al., 2014). The imported raw intensity data were preprocessed by background correction and internal control (Wilhelm-Benartzi et al., 2013). The subset-quantile within array normalization (SWAN) method (Maksimovic et al., 2012) was used to correct the technical bias from the Type I and Type II probe designs (Pidsley et al., 2016). The report of the sample quality control is shown in Supplementary Figure S1. The quality control of probes was performed by filtering probes with missing values or with a detection *p*-value of >0.05 in >25% of the samples (Bibikova et al., 2011). We further removed probes identified by Chen et al. to cross-hybridize with nontargeted DNA (Chen et al., 2013). Finally, 822,246 DNA methylation loci passed quality control. The annotation was performed utilizing the human reference genome GRCh37/hg19.

### Statistical analysis

Differentially methylated DNA loci were identified by linear regression modeling with empirical Bayes approach for standard deviation estimation using the limma package (Ritchie et al., 2015). Significance levels were adjusted to control for the false discovery rate (FDR) using the Benjamini–Hochberg procedure. The temporal pattern (24 h vs. 12 h) was taken into consideration and incorporated into the final identification of differentially methylated DNA loci. Detailed information on the linear regression model and the interpretations of coefficients can be found in Supplementary Material S1 and Supplementary Figure S2.

M-values served as the chosen DNA methylation levels (the dependent variable) in the linear regression model due to their stronger alignment than beta values with the Gaussian assumption, rooted in a Bayesian Gaussian model (Zhuang et al., 2012). The calculation of the M-value involves deriving the log2 ratio between the intensities of methylated *versus* unmethylated probes (Du et al., 2010). A positive M-value signifies a higher count of methylated molecules compared to unmethylated ones, whereas a negative M-value indicates the opposite. However, to facilitate intuitive biological interpretation, the M-values of the identified loci were then transformed into beta values (ranging from 0% to 100%). This transformation directly presents the DNA methylation level as a percentage for a specific locus. The conversion followed this relationship: M = log_2_ (beta/[1–beta]).

Pathway analyses were performed with both the first generation over-representation analysis (ORA) and the second generation functional class scoring (FCS) analysis. For ORA, genes corresponding to the identified DNA methylation loci were used in the web-accessible bioinformatic tool DAVID (Huang da et al., 2009). By gene-set enrichment analysis (GSEA), the DAVID aimed at systematically extracting biological meaning and over-represented biological functions from large gene or protein lists based on the hypergeometric (Fisher’s exact) test. For FCS, all genes were ranked using *p*-values from the differential expression test. By using R Bioconductor package methylGSA (Ren and Kuan, 2019), the entire gene list was utilized to calculate a gene set level *p*-value, with a crucial adjustment made for the number of CpGs to account for the specific biases in EWAS arising from diverse CpG association *p*-values per gene. The gene sets were defined utilizing the KEGG (Kanehisa and Goto, 2000) and GO (Ashburner et al., 2000) database.

To replicate our findings, we tested the identified DNA methylation loci in an external global DNA methylation dataset (generated on Infinium MethylationEPIC platform) from the heart biopsy samples of 41 dilated cardiomyopathy (DCM) and 31 control individuals who underwent heart transplantation (Meder et al., 2017). In order to extend our findings to the population level, we further compared our results to an external epigenomic dataset consisting of blood samples from 1,246 individuals (Shi et al., 2021). In this dataset, physical activity serves as a physiological stimulus analogous to IGF-1 at the population level, while cardiovascular disease represents the pathological changes associated with AngII.

PCR results were tested using the one-way ANOVA followed by the Holm-Sidak post-hoc test to examine differences among groups to control for multiple testing. Statistical significance was set at *p* < 0.05 for two-sided tests.

## Results

### Epigenome-wide association study of cardiomyopathy

**FIGURE 1:**
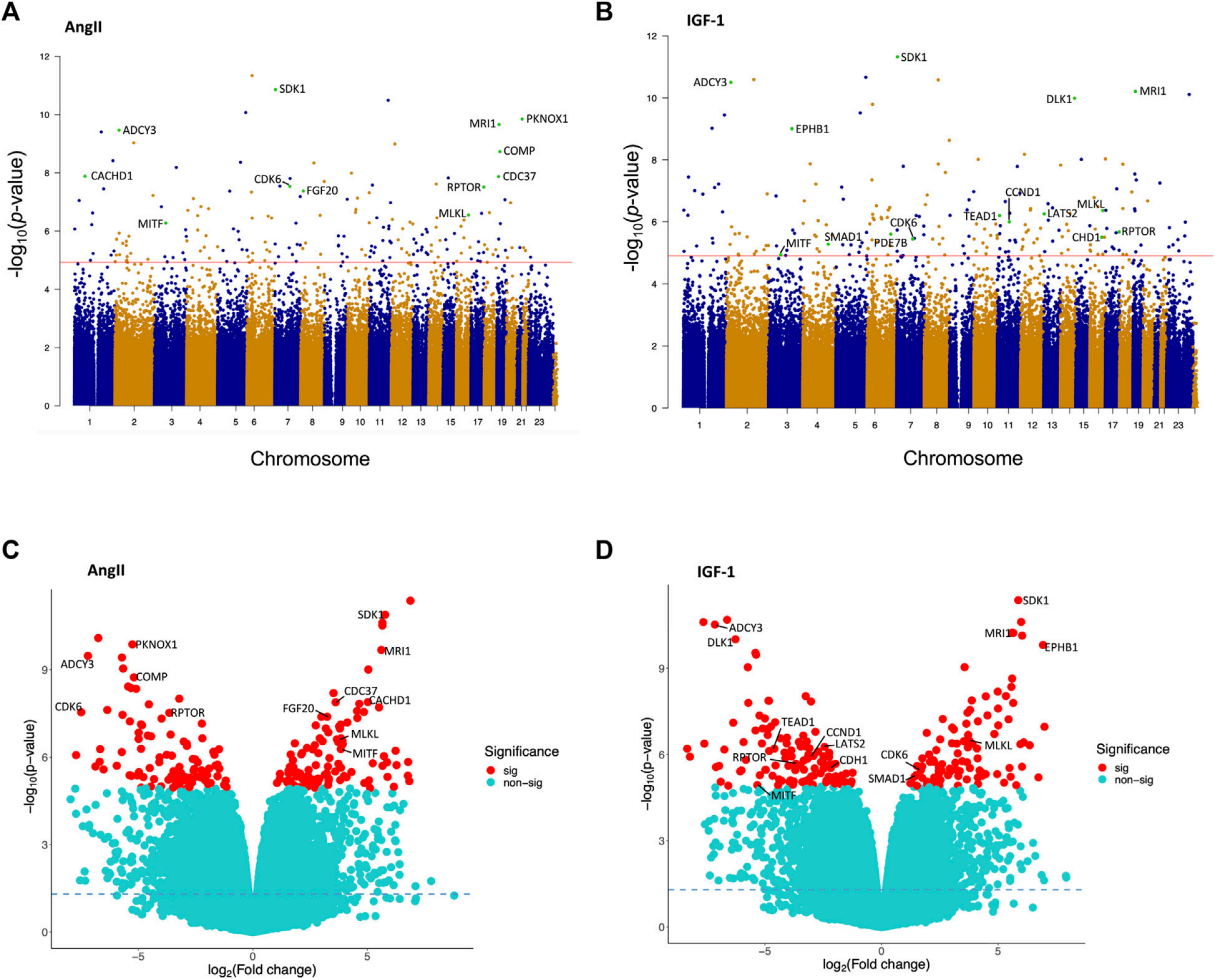
Manhattan plots and volcano plots illustrating the findings of the epigenome-wide association study conducted on human cardiomyocytes post AngII and IGF-1 treatment (vs. control). Manhattan plots are shown individually for **(A)** AngII and **(B)** IGF-1. These plots feature the minus log_10_ *p*-values of DNA methylation loci that have met quality control criteria. The red line represents the significance threshold for the false discovery rate (FDR) at *p* = 0.05. Some of the CpG loci are highlighted as cyan dots and are accompanied by labels indicating their corresponding gene names. Volcano plots are displayed for **(C)** AngII and **(D)** IGF-1. In these plots, DNA methylation loci are denoted as red dots (significant loci) or cyan dots (non-significant loci), according to the FDR significance threshold of *p* = 0.05. Hypermethylated loci are positioned on the right side, while hypomethylated loci are situated on the left side. The horizontal dashed line signifies the unadjusted significance level of *p* = 0.05. Some of the CpG loci the significant CpG loci are annotated with their corresponding gene names. The correspondences between the gene names and the highlighted CpG loci are: *CDK6* (cg03330377 in AngII, cg06576484 in IGF-1), *RPTOR* (cg02251850 in AngII, cg02251850 in IGF-1), *MITF* (cg10160567 in AngII, cg13685139 in IGF-1), *MLKL* (cg08357850), *SDK1* (cg07926598), *PKNOX1* (cg15157241), *MRI1* (cg25755428), *ADCY3* (cg20227471), *COMP* (cg25497529), *CACHD1* (cg16487794), *CDC37* (cg00876541), *FGF20* (cg05578055), *DLK1* (cg17412258), *EPHB1* (cg19591512), *PDE7B* (cg13864354), *LATS2* (cg00580291), *TEAD1* (cg01482376), *CCND1* (cg02723533), *CDH1* (cg08616585), *SMAD1* (cg14561362).

As summarized in the Manhattan plots (Figures 1A, B), and following correction for multiple testing, a total of 194 loci exhibited significant differential methylation in the AngII treatment group (vs. control; refer to Figure 1A), along with 206 significant loci in the IGF-1 treatment group (vs. control; refer to Figure 1B). The volcano plots (Figures 1C,D) depict the distribution of these significant loci (FDR <0.05; represented by red dots) in the sections corresponding to hypermethylation and hypomethylation. After AngII treatment, we observed 97 (50.0%) loci displaying hypermethylation and 97 showing hypomethylation (Figure 1C). Similarly, after IGF-1 treatment, 93 (45.1%) loci exhibited hypermethylation, and 113 demonstrated hypomethylation (Figure 1D). For detailed insights into these significant loci, including their locations, relations to CpG islands, functional regions, corresponding genes, and *p*-values, please refer to Supplementary Table S1. Notably, a majority of these significant loci were situated within gene body regions, closely followed by the promoter regions.

81 DNA methylation sites were shared between both treatment groups (Supplementary Table S2), while 113 loci were unique to the post-AngII treatment scenario, and 125 loci were unique to the post-IGF-1 treatment scenario. Noteworthy CpG loci were highlighted in both Manhattan and Volcano plots due to their corresponding genes being among the most significant in the AngII and IGF-1 groups (e.g., *SDK1*, *MRI1*, *ADCY3*, *etc.*), or due to their recognized roles as pivotal components within important pathways (e.g., *RPTOR* in the mTOR pathway, *LATS2* and *TEAD1* in the Hippo signaling pathway, *CDK6* in the cell cycle, *etc.*) (Figures 1A–D).

### Comparison of significant DNA methylation loci related genes in AngII vs. IGF-1 treatment

**FIGURE 2:**
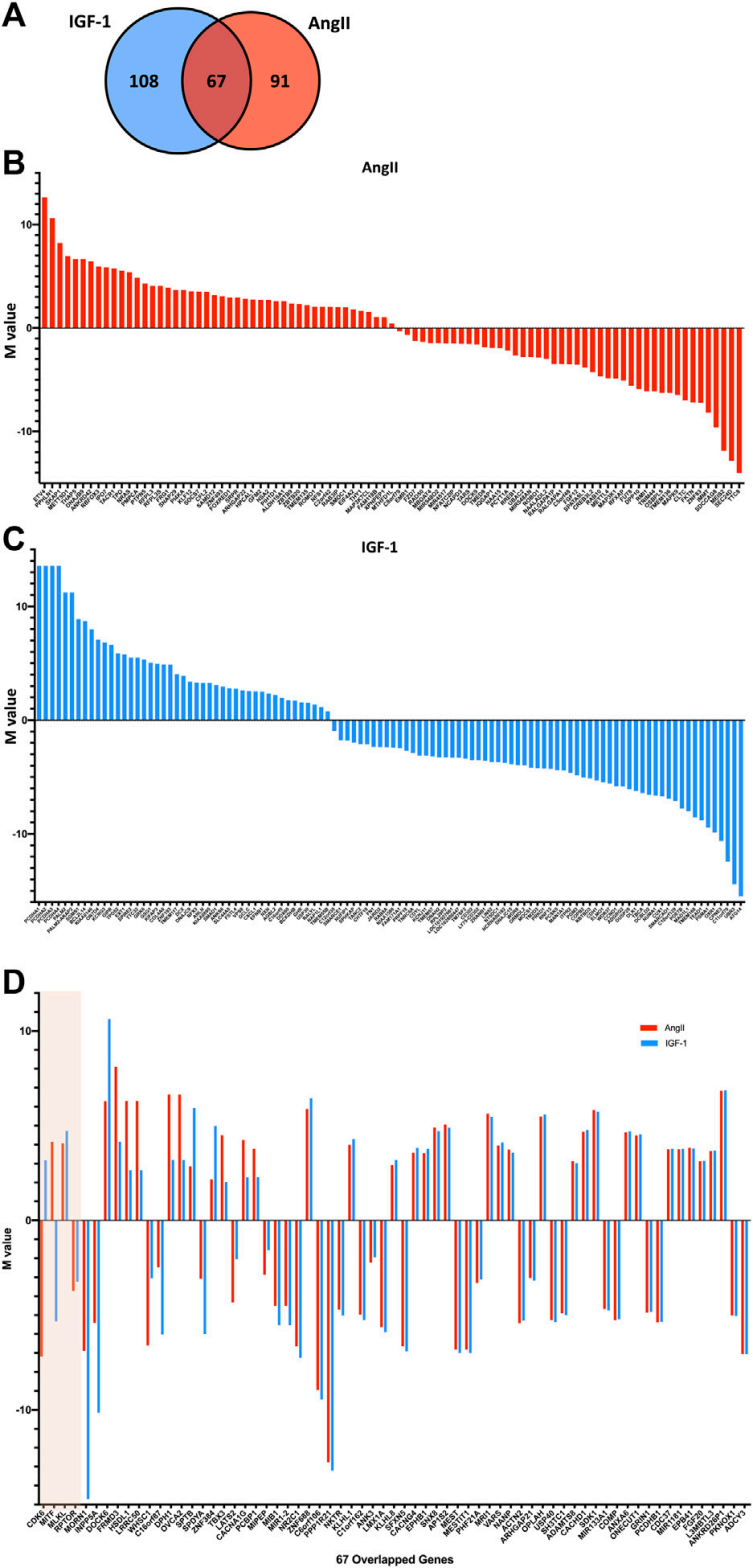
Mapping genes to the significant DNA methylation loci post AngII and IGF-1 treatment (vs. control). The significant DNA methylation loci were annotated to their corresponding genes. Illustrated by a Venn diagram **(A)**, this visualization presents the count of genes annotated from the identified loci in the AngII group (depicted by the red circle), the count of genes annotated from the loci in the IGF-1 group (represented by the blue circle), as well as the overlap between the two. The genes, ordered according to the M-values of their respective significant loci, are presented in **(B)** for those exclusive to AngII and in **(C)** for those exclusive to IGF-1 (shown as blue bars). The solid line signifies the M-value = 0, with hypermethylated loci positioned above the line and hypomethylated loci positioned below. In **(D)**, the 67 overlapping genes are detailed and ranked by delta-delta change. The beige-shaded portion delineates genes (i.e., *CDK6*, *MITF*, *MLKL*, and *RPTOR*) with differing significant loci for AngII and IGF-1, while the unshaded region encompasses genes sharing the same significant locus for both treatments.

Assigning the significant loci to their respective genes and excluding the loci that are not situated near any known genes (Supplementary Table S1), we identified 158 genes corresponding to significant DNA methylation loci after AngII treatment and 175 genes after IGF-1 treatment, with 67 genes overlapped in both AngII and IGF-1 groups (see the Venn diagram Figure 2A). Of the 158 genes identified in the AngII group, 81 (51.3%) genes were related to a significantly hypermethylated loci and 77 genes to a significantly hypomethylated loci. For IGF-1, 80 (45.7%) genes were hypermethylated and 95 genes were hypomethylated. The 91 genes which are exclusively identified after AngII treatment are shown in Figure 2B and ranked by the M-values of their corresponding significant loci, while the 108 genes which are exclusive for IGF-1 treatment are shown in Figure 2C. These distinct genes in both treatments could significantly contribute to the variations in mechanisms between physiological and pathological cardiomyopathy, warranting further investigation.

However, in this study, we narrow our focus to another aspect of mechanism disparities - variations within the same gene. Our aim is to identify genes that might exhibit differential methylation in response to AngII and IGF-1 treatment. These genes hold the potential to serve as targets, as manipulating them could potentially lead to achieving specific goals by regulating one aspect to control the opposing outcomes. For each overlapped gene, we compared the differential change in methylation level between AngII and control (delta change) with the differential change between IGF-1 and control (delta change), resulting in a delta-delta change. The absolute value of the delta-delta change reflects the extent to which the same gene’s methylation status changed differently after AngII vs. IGF-1 treatment. The 67 overlapped genes are presented in Figure 2D, ordered based on the absolute values of the delta-delta change.

Among these overlapped genes, four genes (*CDK6*, *MITF*, *MLKL*, and *RPTOR*), shown in the shaded area of Figure 2D, are unique because the corresponding significant loci differ between the AngII and IGF-1 groups. For *CDK6*, the CpG site cg03330377 (location chr7: 31232748) showed significant hypomethylation after AngII treatment, while cg06576484 (location chr7: 92382242) exhibited significant hypermethylation after IGF-1 treatment. Similarly, for *MITF*, cg10160567 (location chr3: 69788520) demonstrated significant hypermethylation after AngII treatment, while cg13685139 (location chr3: 69812820) displayed significant hypomethylation after IGF-1 treatment. In the case of *MLKL*, cg08357850 (location chr16: 74734885) was identified as significant in the AngII group, while both cg09830308 (location chr16: 74734321) and cg08357850 (location chr16: 74734885) were significant in the IGF-1 group, all showing hypermethylation. Similarly, for *RPTOR*, cg02251850 (location chr17: 78851503) was significant in the AngII group, while both cg02243479 (location chr17: 78859959) and cg02251850 (location chr17: 78851503) were significant in the IGF-1 group, all displaying hypomethylation.

### Pathway analysis and validations using data from human samples

Pathways were identified through gene-set enrichment analysis (GSEA) using genes corresponding to differentially methylated loci identified in the AngII and IGF-1 treatment groups via the ORA analysis (Table 1). The melanoma pathway showed the highest enrichment in both the AngII and IGF-1 groups [*p* = 0.003 with 8.35-fold enrichment, and *p* = 0.004 with 7.57-fold enrichment, respectively]. Additionally, the PI3K-Akt signaling pathway was among the top enriched pathways for both treatment groups [*p* = 0.023 with 2.75-fold enrichment, and *p* = 0.013 with 2.80-fold enrichment, respectively]. Interestingly, the PI3K-Akt signaling pathway shared all genes enriched in melanoma pathway (e.g., *CDK6, FGF20*, and *MITF*) and included three additional genes (i.e., *RPTOR, COMP, and CDC37*), suggesting a higher level of significance and potential relevance. It is noteworthy that the pathways identified through the FCS analysis supported the outcomes of the ORA analysis by enriching genes within melanoma, mTOR signaling, cell cycle, and MAPK signaling pathways (Supplementary Figure S3). These pathways either overlap, interact with, or form a subset of the PI3K-Akt signaling pathway.

**TABLE 1:** Gene-set enrichment analysis of genes identified by AngII and IGF-1 related DNA methylation loci.

Term	Count	*p*-value	Genes	Fold Enrichment
AngII				
Melanoma	5	0.003	***CDK6*** *, FGF12,* ***FGF20*** *,* ***MITF*** *, NRAS*	8.35
MAPK signaling pathway	8	0.005	*CACNG4,* ***CACNA1G*** *, FGF12,* ***FGF20*** *, MAPK9, MAP3K1, NRAS, PTPN5*	3.75
Melanogenesis	5	0.009	***ADCY3*** *, CREB3L2, FZD7,* ***MITF*** *, NRAS*	5.93
PI3K-Akt signaling pathway	8	0.023	*CREB3L2,* ***COMP*** *,* ***CDC37, CDK6*** *, FGF12,* ***FGF20*** *, NRAS,* ***RPTOR***	2.75
Rap1 signaling pathway	6	0.029	***ADCY3*** *, FGF12,* ***FGF20*** *, GRIN1, NRAS, SKAP1*	3.39
Regulation of actin cytoskeleton	6	0.029	*IQGAP1, ACTN2, CFL2, FGF12, FGF20, NRAS*	3.39
Hepatitis B	5	0.032	*CREB3L2,* ***CDK6*** *, MAPK9, MAP3K1, NRAS*	4.09
GnRH signaling pathway	4	0.039	***ADCY3*** *, MAPK9, MAP3K1, NRAS*	5.21
Pathways in cancer	8	0.042	***ADCY3*** *,* ***CDK6*** *, FGF12,* ***FGF20*** *, FZD7,* ***MITF*** *, MAPK9, NRAS*	2.41
Axon guidance	4	0.088	*EPHB1, CFL2, NRAS, ROBO1*	3.74
**IGF-1**				
Melanoma	5	0.004	***CDK6*** *,* ***FGF20*** *, CDH1, CCND1,* ***MITF***	7.57
PI3K-Akt signaling pathway	9	0.013	*COL4A6,* ***COMP*** *,* ***CDC37*** *,* ***CDK6*** *,* ***FGF20*** *,* ***RPTOR*** *, CCND1, FN1, GHR*	2.80
Aldosterone synthesis and secretion	4	0.038	***ADCY3*** *,* ***CACNA1G*** *, ITPR2, PRKD1*	5.31
Signaling pathways regulating pluripotency of stem cells	5	0.039	*SMAD1, SMARCAD1, TBX3, JARID2, ONECUT1*	3.84
Small cell lung cancer	4	0.043	*COL4A6, CCND1,* ***CDK6*** *, FN1*	5.06
Hippo signaling pathway	5	0.049	*SMAD1, TEAD1, CDH1, CCND1, LATS2*	3.56
Pathways in cancer	8	0.066	***ADCY3*** *, CDH1, COL4A6, CCND1,* ***CDK6*** *,* ***FGF20*** *, FN1,* ***MITF***	2.19

Genes in bold indicate the genes identified in both AngII and IGF-1 groups.

**FIGURE 3:**
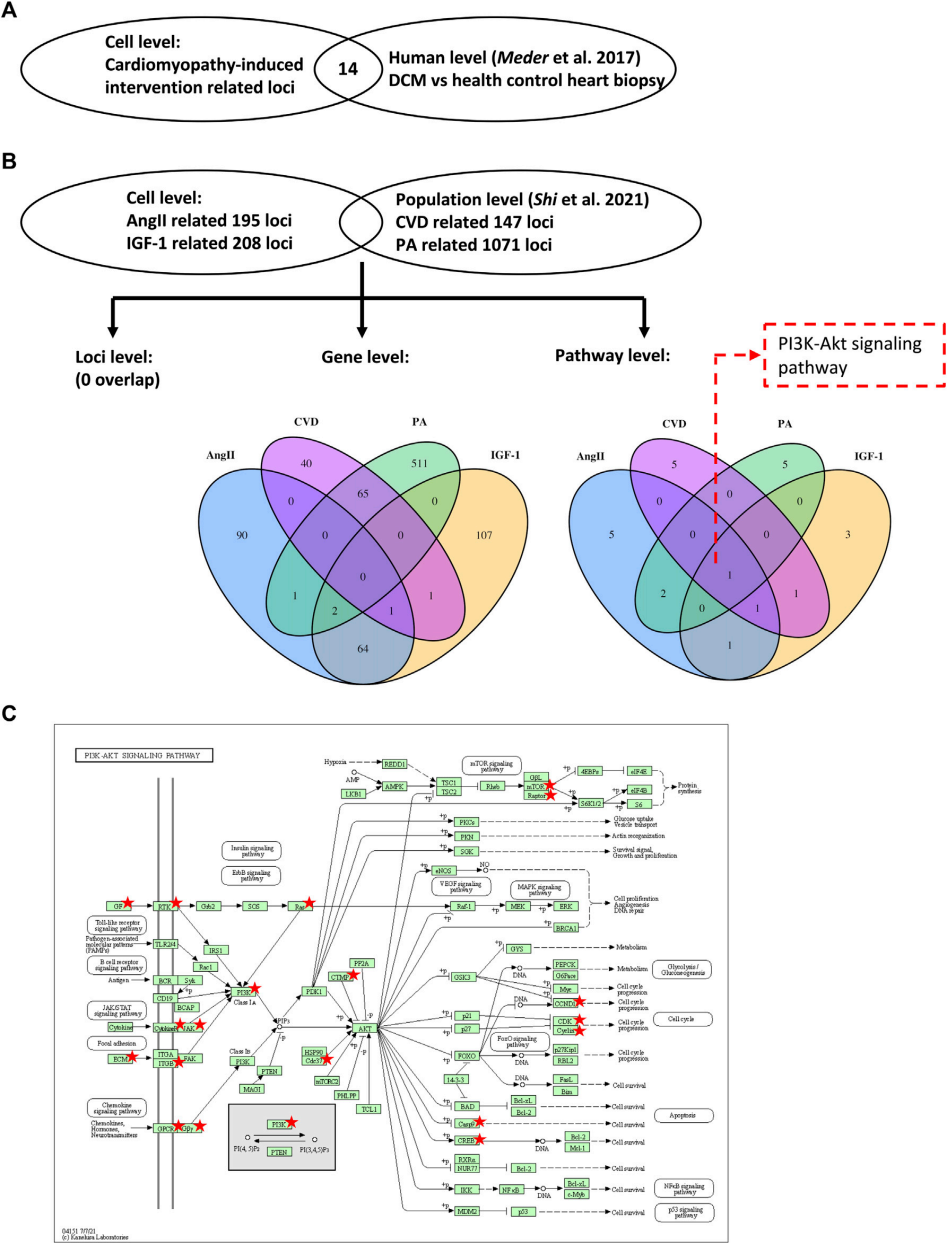
Validations using data from human samples. Two external datasets using human heart biopsy samples (Meder et al., 2017) and human blood samples (Shi et al., 2021) were selected to validate the results from the present study. Venn diagrams illustrate the overlap of identified loci **(A)**, genes and pathways **(B)**. The KEGG map **(C)** displays the PI3K-Akt pathway, with the identified genes marked by stars. DCM, dilated cardiomyopathy; CVD, cardiovascular diseases; and PA, physical activity. KEGG, Kyoto Encyclopedia of Genes and Genomes. Copyright permission was granted from Kanehisa Laboratories (https://www.kanehisa.jp/).

To replicate our findings, we tested the identified DNA methylation loci in an external global DNA methylation dataset from the heart biopsy samples of 41 DCM and 31 healthy control individuals (Meder et al., 2017). As shown in Figure 3A; Supplementary Table S3, we could successfully replicate 14 of the 319 loci (4.4%), corresponding to 12 of the 266 (4.5%) mapped genes in the independent dataset. Two of the most stringently significant hits (i.e., cg16967640, cg07202461) from our findings could also be validated (replication adjusted *p* = 0.021 and 0.046, respectively).

To extend our findings from the cell level to the population level, we compared our findings to an external epigenomic dataset from the blood samples of 1,246 individuals (Shi et al., 2021). In this cohort study, the physical activity- (PA) and CVD-associated DNA methylation loci were identified. At the CpG site level, no locus was mutually identified in our study and in the independent cohort. At the gene level, two genes (i.e., *EPB41* and *MORN1*) were mutually identified associated with both IGF-1 treatment (in our study) and PA (in the cohort study) (Figure 3B). One gene (i.e., *CACHD1*) was mutually identified associated with both AngII treatment (in our study) and CVD (in the cohort study) (Figure 3B). Interestingly, these three mutual genes did not exhibit a significant change in mRNA expression during the confirmatory step (Supplementary Figure S4). Consequently, we shifted our focus to exploring overlaps at the pathway level. Pathway analysis of the cohort study is shown in Supplementary Table S4.

Notably, we observed enrichment of the PI3K-Akt signaling pathway across all investigated conditions (i.e., AngII, IGF-1, PA, and CVD) at the pathway level, further supporting our previous findings indicating its significant role. In Figure 3C, the PI3K-Akt signaling pathway is illustrated, with the genes identified in our study and the cohort study highlighted. Remarkably, among the highlighted genes, *CDK6* and *RPTOR* emerge as key factors within this pathway, providing additional support to our previous observations where the DNA methylation levels of *CDK6* and *RPTOR* exhibited differential changes following AngII and IGF-1 treatment.

### Impact of differential DNA methylation on gene expression

**FIGURE 4:**
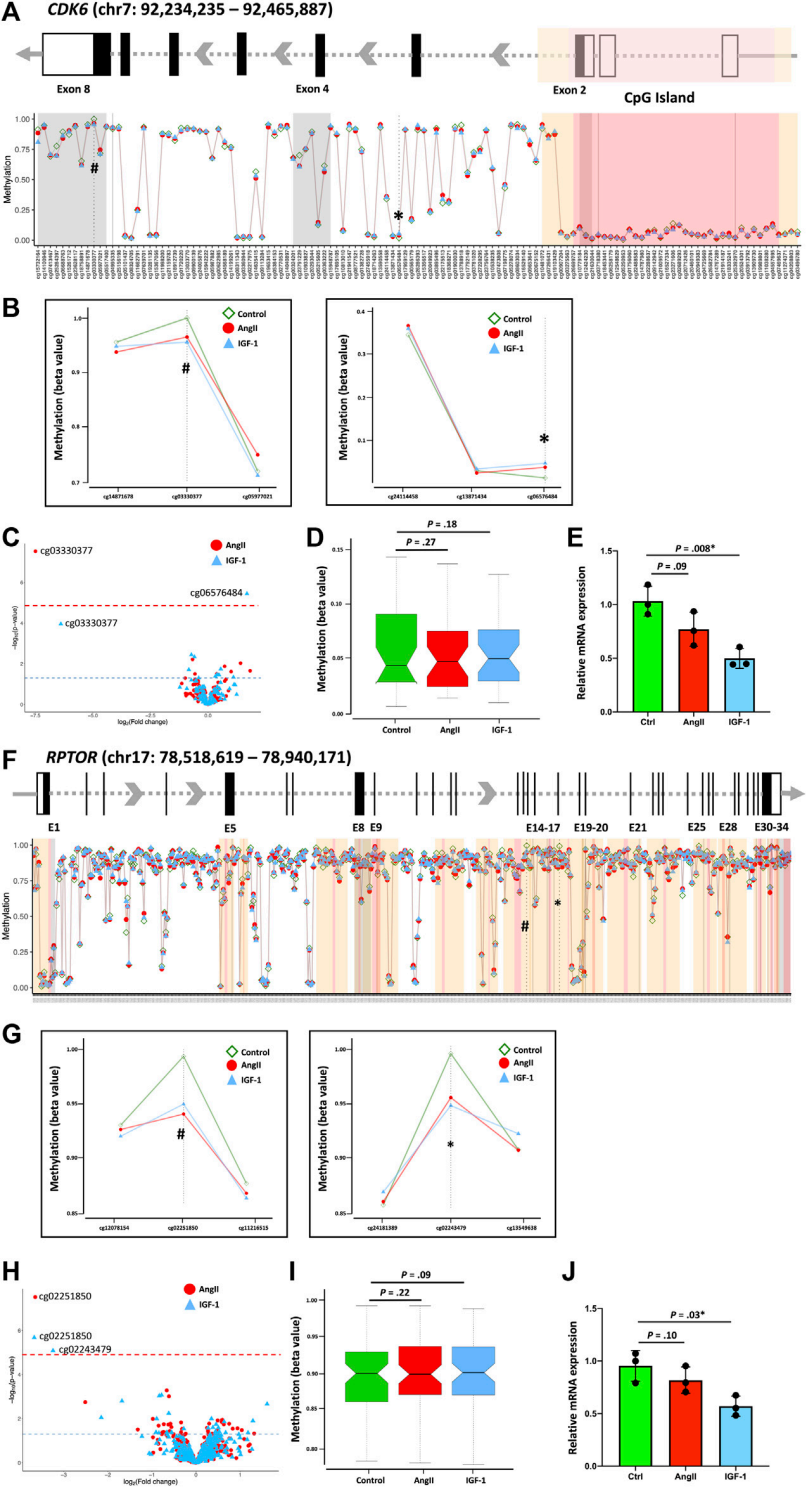
Association of DNA methylation pattern and gene expression. The association of DNA methylation pattern and gene expression were displayed using *CDK6* and *RPTOR* as examples. Schematic gene structures **(A, F)** display exons (black boxes or bars) and the 3′-UTR and 5′-UTR regions (empty boxes). The distances between exons are not depicted to scale but are modified for visual representation of the DNA methylation loci-enriched region corresponding to the global methylation distribution map across the *CDK6* **(A)** and *RPTOR* **(F)** genes. The red-shaded area indicates the CpG island, while the yellow-shaded areas on both flanks indicate CpG shelves and CpG shores. The identified significant loci’s DNA methylation levels are indicated in the global map and presented in separate Figures. For *CDK6*, the loci are cg03330377 and cg06576484 **(B)**, and for *RPTOR*, the loci are cg02251850 and cg02243479 **(G)**. Volcano plots present all CpGs within the *CDK6* gene **(C)** and the *RPTOR* gene **(H)** following AngII and IGF-1 treatments. The red dashed line indicates the FDR significance threshold of *p* = 0.05, utilized to adjust for the complete set of 822,246 DNA methylation loci that passed quality control. Hypermethylated loci are positioned on the right, while hypomethylated loci are on the left. The blue horizontal dashed line represents the unadjusted significance level of *p* = 0.05. Boxplots display the average methylation level of the CpG island(s) for *CDK6* **(D)** and *RPTOR* **(I)**. Gene expression levels are shown in **(E)** for *CDK6* and in **(J)** for *RPTOR*.

To investigate whether the identified DNA methylation alterations also impact overall gene expression, we conducted qPCR on isolated RNA extracts for specific genes within the PI3K-Akt pathway. Notably, the expression of eight genes was found to correlate with changes in DNA methylation sites (Figure 4; Supplementary Figure S5). Upon analyzing the methylation status of the *CDK6* gene, we observed substantial hypomethylation in the CpG island and flanking shores and shelves regions, yet notable hypermethylation within the gene body region (Figure 4A). Within this context, we identified two distinct differentially methylated sites for AngII (cg03330377, within the 3′UTR) and for IGF-1 (cg06576484, within the intron) treatments (Figure 4B). Interestingly, the CpG locus cg03330377 displayed discernible hypomethylation in IGF-1 treatment among all *CDK6* CpG loci, with a *p*-value (1.1 × 10^−4^) that approached but did not reach the global FDR = 0.05 cutoff line (Figure 4C). While the average DNA methylation level of the CpG island indicated a tendency toward increased methylation in both AngII and IGF-1 groups compared to the control group, the difference was not statistically significant (Figure 4D). Remarkably, despite hypermethylation or hypomethylation of significant CpGs, the *CDK6* gene exhibited reduced expression in both the AngII and IGF-1 groups, with significance observed solely in the IGF-1 group (Figure 4E). Given the absence of a significant change across the overall CpG island, it is plausible that both cg03330377 and cg06576484 are linked to a decrease in gene expression, with their combined presence contributing to the substantial decrease observed in the context of IGF-1 treatment. Although the exact function of gene body DNA methylation in gene transcription remains unclear, prior studies suggest that gene body DNA methylation might be a general feature, and contribute to transcriptional regulation (Hellman and Chess, 2007; Ball et al., 2009).

A parallel scenario emerged in the case of the *RPTOR* gene (Figure 4F). We identified two distinct differentially methylated sites for AngII (cg02251850, within the CpG shores) and for IGF-1 (cg02251850 and cg02243479, both within the CpG shores) treatments (Figures 4G,H). Similarly, the average DNA methylation level of the CpG island showed a trend toward increased methylation in both the AngII and IGF-1 groups in comparison to the control group, yet the difference lacked statistical significance (Figure 4I). The *RPTOR* gene also displayed reduced expression in both the AngII and IGF-1 groups, with significance exclusively observed in the IGF-1 group (Figure 4J). It is conceivable that cg02251850 and cg02243479 collectively contribute to a decrease in gene expression, leading to the notable decrease observed in the context of IGF-1 treatment.

However, our study did not delve into the mechanisms by which these CpG loci regulate gene expression. It remains a viable hypothesis that these highlighted CpG loci might be situated within pivotal regulatory elements, warranting further investigation in future studies.

## Discussion

The present *in vitro* study identified a significant role of DNA methylation patterns and multiple related pathways in the pathological and physiological hypertrophic processes. The reproducible DNA methylation sites and the PI3K-Akt signaling pathway identified in this study, as well as the successful replications in external human biopsy samples and population blood samples, underscored the robustness of the findings.

The PI3K-Akt signaling pathway has been demonstrated to be involved in both physiological and pathological forms of cardiac growth. PI3K has been shown to activate in response to pressure overload *in vivo* and in response to hypertrophic ligands such as growth factors, angiotensin II, and cardiotrophin-1 *in vitro* (Oh et al., 1998). Overexpression of the activated PI3K can directly bind to the pleckstrin homology domain of Akt and promote translocation and indirectly activate Akt by activating the kinase that phosphorylates Akt (Walsh, 2006). In turn, Akt activation regulates cardiomyocyte size through downstream pathways such as activation of mTOR-dependent pro-growth pathways and cyclin/CDK cell cycle regulation, and suppression of FOXO-dependent atrophy programs. Our previous study identified an important catalytic subunit of PI3K (i.e., *PIK3CD*) that is significantly hypomethylated in physically active individuals and hypermethylated in CVD patients, indicating the compensation of the PI3K-Akt signaling pathway at the DNA methylation level in response to long-term physiological and pathological stresses to hearts (Shi et al., 2021). In contrast, in our present study, we did not find significant changes of either PI3K or Akt at DNA methylation levels in response to AngII or IGF-1 treatment on cardiomyocytes. This result suggests that the alteration of upstream molecules in the PI3K-Akt signaling might be regulated by other ways (e.g., direct protein-protein interaction) rather than DNA methylation in the *in vitro* model mimicking physiological or pathological stresses to cardiomyocytes.

However, some downstream key components of the PI3K-Akt pathway were identified to have significant changes at the DNA methylation level in response to AngII or IGF-1 treatment. *CDK6*, as an example of a cyclin/CDK cell cycle regulator, showed significant changes on different DNA methylation loci in response to AngII vs. IGF-1. The gene expression confirmed the opposite change of *CDK6* corresponding to DNA methylation modifications. A prior study showed that *CDK6* is expressed in the adult left ventricle of the heart and is activated by D-type cyclins (Busk et al., 2002). Tamamori et al. (Tamamori et al., 1998) and Busk et al. (Busk et al., 2002) showed that inhibition of *CDK6* impaired hypertrophy of *in vitro* isolated cardiomyocytes. MicroRNA-1 was also reported to be involved in the regulation of *CDK6* mediated cardiac hypertrophy (Yuan et al., 2016). Our study provided new evidence of a possible mechanism, that AngII or IGF-1 may affect *CDK6* methylation to regulate the cell cycle program. Similarly, *RPTOR*, as an example of mTOR-dependent growth regulator, was identified to have different regulation loci in response to AngII vs. IGF-1 at the DNA methylation level. Recent *in vivo* studies demonstrated that *RPTOR* plays a critical role in hypertrophy (Shende et al., 2011; Baraldo et al., 2021). Shende et al. showed that cardiac ablation of Raptor protein encoding by *RPTOR* impairs adaptive hypertrophy and causes dilated cardiomyopathy and heart failure in mice (Shende et al., 2011). Our study indicates an alternative way to activate mTOR signaling by regulating the DNA methylation status of *RPTOR*.

In additional to the well-established PI3K-Akt signaling pathway, our study identified many DNA methylation loci and genes with different methylation levels in response to AngII vs. IGF-1 (Figure 2). Some identified genes and their related pathways have recently received significant attention in the field of cardiac biology to potentially differentiate between physiological and pathological cardiac hypertrophy. For example, *CCND1, LATS2, CDH1, TEAD1*, and *SMAD1* are all important components of the Hippo signaling pathway, which is shown to be a critical regulator of heart growth (Windmueller and Morrisey, 2015). One recent study suggested that the Hippo pathway is involved in both physiological cardiac hypertrophy (Gholipour and Tabrizi, 2020) and can improve the heart’s response to CVD (Yang et al., 2015). Interestingly, Lin et al. reported that *PI3KCB*, which encodes another important component of PI3K kinase, links Hippo and PI3K-Akt signaling pathways to promote heart growth (Lin et al., 2015). In the present study, *CCND1, LATS2, CDH1, TEAD1*, and *SMAD1* were all significantly hypomethylated in response to IGF-1, but not to AngII. Confirmed by RT-PCR, gene expression of *CCND1, LATS2, CDH1*, and *TEAD1* were all significantly decreased in IGF-1 but remained unchanged in AngII (Supplementary Figure S6). In agreement with previous reports, our findings indicated the Hippo signaling pathway may be more active in response to IGF-1 but not to AngII. Further study is promising for discovering the possible role of the Hippo signaling pathway in differentiating between pathological and physiological cardiac hypertrophy.

We noticed some limitations in the present study. First, the biomaterials of the study were extracted from the cell line AC16. While AC16 cells were derived from normal adult human ventricular cardiomyocytes, they were fused with SV40 transformed fibroblasts to gain immortality. Nevertheless, AC16 remains a crucial *in vitro* model for uncovering key aspects of cardiomyopathy mechanisms (Chen et al., 2020). Secondly, it is important to note that the samples used in our present cell line study differ from those in our previous population study. The population study utilized monocytes derived from blood samples, whereas the current study focused on cardiomyocytes. This distinction could explain the lack of overlapping loci identified when comparing the cell study to the population study. However, obtaining biopsy samples for a large population is not feasible, and considering that cardiomyopathy involves both cardiomyocytes and inflammatory cells, it is known that inflammatory signaling in cardiomyocytes responds to stress (Xu and Brink, 2016). Therefore, we believe that this comparison can still provide valuable insights into the inflammatory signaling response to pathological and physiological stimuli. Lastly, we examined the impact of DNA methylation on gene expression through confirmatory PCR analysis. Conventionally, it is believed that DNA methylation suppresses gene expression. However, emerging evidence suggests that DNA methylation can also enhance gene expression in certain cases (Moore et al., 2013). Additionally, several regulatory steps occur between DNA methylation and gene transcription. For instance, a complex interplay (i.e., cooperation or antagonism) exists between DNA methylation and histone modifications, directly influencing the final gene expression outcome (Rose and Klose, 2014). However, these intricacies are not addressed within the scope of this study. Therefore, to gain a comprehensive understanding of the identified genes, further investigations into their biological roles through mechanistic research are warranted.

## Conclusion

Our study was among the first to profile the whole-genome level of DNA methylation changes in adult human cardiomyocytes in response to pure pathological stress (i.e., AngII) and pure physiological stress (i.e., IGF-1). Validated by external human heart biopsy data, compared with a cohort study, and confirmed by RT-PCR experiments, the identified DNA methylation loci, genes, and pathways might have the potential to differentiate between the pathological vs. physiological cardiac hypertrophy.

## Data Availability

The data presented in this study are deposited in the Gene Expression Omnibus (GEO) under the accession number GSE241551.

## Abbreviations

AngII, Angiotensin II; IGF-1, Insulin-like growth factor 1; PA, Physical activity; CVD, Cardiovascular diseases; DCM, Dilated cardiomyopathy; GSEA, Gene-set enrichment analysis.
