# Supplementary material for: Epigenome-wide DNA methylation profiling in comparison between pathological and physiological hypertrophy of human cardiomyocytes
Source: Front Genet. 2023 Sep 27;14:1264382. doi: 10.3389/fgene.2023.1264382 (PMC10565041; doi:10.3389/fgene.2023.1264382)
Supplement: Supplementary file 1 [file DataSheet1.docx]

**Supplementary materials**

**Supplementary methods**

**Table S1. Detailed information of the significant loci.**

**Table S2. Detailed information of the overlapped loci.**

**Table S3. Replicated DNA methylation loci.**

**Table S4. Pathway analysis of the cardiovascular diseases (CVD) and physical activity (PA) related DNA methylation loci based on the dataset form the MESA study.**

**Figure S1. Sample quality control report.**

**Figure S2. Visualized interpretation of conditions categorized by the three linear regression coefficients.**

**Figure S3. Pathway analysis conducted through the functional class scoring methods using both KEGG and GO databases.**

**Figure S4. Validation of gene expressions for the thee mutual genes in both the current study and the cohort study.**

**Figure S5. Global DNA methylation distribution and gene expressions of some selected genes in PI3K-Akt pathway.**

**Figure S6. Validation of gene expressions of some selected genes in Hippo signaling pathway.**

**Supplementary methods**

**Linear regression model:**

For each DNA methylation locus, the linear regression model was fitted separately for AngII and IGF-1:

$$Y_{i}=\beta_{0}+\beta_{1}X_{i1}+\beta_{2}X_{i2}+\beta_{3}X_{i1}X_{i2}+\varepsilon_{i}$$

where, for $i$ = 1, …, n observations: $Y_{i}$ denotes the DNA methylation level for $i_{\mathrm{th}}$ DNA methylation locus; $X_{i1}$, and $X_{i2}$ are exploratory variables, where $X_{i1}$ is the treatment effect of Angll or IGF-1 compare to the control for $i_{\mathrm{th}}$ DNA methylation locus, $X_{i2}$ is exposure time using 12h as the reference group (i.e., 12h vs 24h), and $X_{i1}X_{i2}$ is the interaction between treatment and time.

For the linear model used in this study, three coefficients (i.e., $\beta_{1}$ for treatment [AngII or IGF-1 vs control], $\beta_{2}$ for time interval [12h or 24h], and $\beta_{3}$ for the interaction between treatment and time) were used for screening significantly changed DNA methylation loci, resulting in seven possible combinations of the three coefficients (i.e., $\beta_{1}\beta_{2}\beta_{3},\beta_{1}\beta_{2},\beta_{2}\beta_{3},\beta_{1}\beta_{3},\beta_{1},\beta_{2} and \beta_{3}$) for corresponding conditions (See **Supplementary Figure 2A**). The number of significantly differential DNA methylation loci for each condition and their overlaps were shown in **Supplementary Figure 2B** (for AngII) and **2C** (for IGF-1). The majority of the significant loci were identified in three combinations/conditions. They are 1) $\beta_{1}\beta_{2}\beta_{3}$, indicating that the methylation level of one specific locus changed over time in both treatment and control groups with different degrees; 2) $\beta_{1}\beta_{2}$, indicating that the DNA methylation level of one specific locus changed over time in both treatment and control groups with the same degree but different basal levels; 3) exclusive $\beta_{1}$, indicating that the DNA methylation level of one specific locus did not change over time in either treatment or control group, but the basal methylation level were different in the two groups. The condition where only $\beta_{2}$ was significant (i.e., indicating that the DNA methylation level of one specific locus changed over time in both treatment and control groups with the same degree and the same basal level) was excluded from the screening pool due to the lack of a significant effect from the treatment component.

**AngII and IGF-1 treatment duration:**

Cardiomyocyte hypertrophic remodeling can be triggered by exposure to AngII or IGF-1 for a duration of at least 12 hours, as evidenced by prior studies and our preliminary findings. Watkins et al. induced cardiomyocyte hypertrophy *in vitro* using 200 nM AngII for 4 hours and observed alterations in gene expression (Watkins et al., 2012). Similarly, Vinciguerra et al. established an *in vitro* model of cardiomyocyte hypertrophy using 20 ng/ml IGF-1 for 24 hours (Vinciguerra et al., 2009). Drawing from these published accounts and our own insights, we conducted preliminary experiments employing 100 nM AngII and 100 ng/ml IGF-1 to ascertain suitable time points (i.e., 6 h, 12 h, and 24 h) based on changes in hypertrophic marker genes like NPPA. Our observations indicated that marker genes began exhibiting changes at 12 hours and became significantly altered at 24 hours. Consequently, we selected 12 h and 24 h as the time points for our study.

**Primers:**

*COL4A6*

(F) CTTCAGGACCCAAAGGAAAGA (R) CCTTGCCTGGTTCTCCTATTAC

*CREB3L2*

(F) ATGTACCACACGCACTTCTC (R) CCTCCATTGACACACTCTTCTC

*FGF12*

(F) CAGCGACTACACTCTCTTCAATC (R) TCACCATTCATGGCCACATAG

*GHR*

(F) TTGCCCTCAACTGGACTTTAC (R) CTCCAGAACCATCCATCCTTTC

*NRAS*

(F) CAGTGCCATGAGAGACCAATAC (R) TCTGCTCCCTGTAGAGGTTAAT

*FN1*

(F) CCACAGTGGAGTATGTGGTTAG (R) CAGTCCTTTAGGGCGATCAAT

**References:**

VINCIGUERRA, M., SANTINI, M. P., CLAYCOMB, W. C., LADURNER, A. G. & ROSENTHAL, N. 2009. Local IGF-1 isoform protects cardiomyocytes from hypertrophic and oxidative stresses via SirT1 activity. *Aging (Albany NY),* 2**,** 43-62.

WATKINS, S. J., BORTHWICK, G. M., OAKENFULL, R., ROBSON, A. & ARTHUR, H. M. 2012. Angiotensin II-induced cardiomyocyte hypertrophy in vitro is TAK1-dependent and Smad2/3-independent. *Hypertens Res,* 35**,** 393-8.

**Table S1. Detailed information of the significant loci.**

| **Name** | **Chr** | **Position** | **CpG Island** | **Region** | **Related gene** | **P.value** | **FDR** | **Methyl** |
| --- | --- | --- | --- | --- | --- | --- | --- | --- |
| **AngII** | | | | | | | | |
| cg23536255 | chr6 | 31276105 | N_Shore |  |  | 4.56E-12 | 3.75E-06 | HYPER |
| cg07926598 | chr7 | 4175200 | OpenSea | Body | SDK1 | 1.38E-11 | 5.69E-06 | HYPER |
| cg07627556 | chr11 | 115530590 | S_Shore |  |  | 3.22E-11 | 8.82E-06 | HYPER |
| cg18530551 | chr5 | 172670879 | Island |  |  | 8.47E-11 | 1.74E-05 | hypo |
| cg15157241 | chr21 | 44396204 | S_Shore | 5'UTR | PKNOX1 | 1.41E-10 | 2.33E-05 | hypo |
| cg25755428 | chr19 | 13875111 | Island | TSS1500 | MRI1 | 2.18E-10 | 2.98E-05 | HYPER |
| cg20227471 | chr2 | 25065550 | OpenSea | Body | ADCY3 | 3.43E-10 | 4.03E-05 | hypo |
| cg16967640 | chr1 | 165205453 | Island | Body | LMX1A | 3.94E-10 | 4.05E-05 | hypo |
| cg11921348 | chr2 | 116098753 | OpenSea | Body | DPP10 | 9.33E-10 | 8.35E-05 | hypo |
| cg23658477 | chr12 | 22094371 | Island |  |  | 1.02E-09 | 8.35E-05 | HYPER |
| cg25497529 | chr19 | 18901364 | Island | Body | COMP | 1.86E-09 | 0.00013896 | hypo |
| cg15578091 | chr1 | 236908064 | OpenSea | Body | ACTN2 | 3.83E-09 | 0.00026274 | hypo |
| cg04182363 | chr8 | 81316879 | OpenSea |  |  | 4.58E-09 | 0.00026925 | hypo |
| cg12664940 | chr5 | 140430875 | OpenSea | TSS200 | PCDHB1 | 4.34E-09 | 0.00026925 | hypo |
| cg19591512 | chr3 | 134514284 | Island | 5'UTR | EPHB1 | 6.55E-09 | 0.00035921 | HYPER |
| cg05183229 | chr10 | 25012543 | Island | 1stExon | ARHGAP21 | 1.02E-08 | 0.00052569 | hypo |
| cg00876541 | chr19 | 10515578 | Island | TSS1500 | MIR1181 | 1.34E-08 | 0.00061336 | HYPER |
| cg16487794 | chr1 | 64936656 | S_Shore | Body | CACHD1 | 1.31E-08 | 0.00061336 | HYPER |
| cg02389501 | chr7 | 93757948 | OpenSea |  |  | 1.57E-08 | 0.00064468 | hypo |
| cg15628498 | chr15 | 53082902 | Island | TSS1500 | ONECUT1 | 1.50E-08 | 0.00064468 | HYPER |
| cg17301223 | chr8 | 145106438 | Island | Body | OPLAH | 1.97E-08 | 0.00077283 | HYPER |
| cg13285686 | chr14 | 66137361 | OpenSea | Body | FUT8 | 2.43E-08 | 0.00090707 | hypo |
| cg06873166 | chr11 | 18814391 | S_Shore | TSS1500 | PTPN5 | 2.64E-08 | 0.00094311 | HYPER |
| cg01316109 | chr7 | 31232748 | Island |  |  | 2.86E-08 | 0.00095508 | HYPER |
| cg03330377 | chr7 | 92242243 | OpenSea | 3'UTR | CDK6 | 2.90E-08 | 0.00095508 | hypo |
| cg02251850 | chr17 | 78851503 | S_Shore | Body | RPTOR | 3.07E-08 | 0.0009715 | hypo |
| cg02664390 | chr1 | 179911884 | OpenSea |  |  | 3.55E-08 | 0.00108188 | hypo |
| cg05578055 | chr8 | 16859045 | Island | Body | FGF20 | 4.20E-08 | 0.00120145 | HYPER |
| cg21182896 | chr5 | 74062681 | Island | 5'UTR | GFM2 | 4.24E-08 | 0.00120145 | HYPER |
| cg11497372 | chr6 | 27513092 | OpenSea |  |  | 4.59E-08 | 0.00125751 | HYPER |
| cg06139099 | chr10 | 134499505 | OpenSea | Body | INPP5A | 4.82E-08 | 0.00127725 | hypo |
| cg10030268 | chr2 | 234465509 | OpenSea | ExonBnd | USP40 | 5.99E-08 | 0.00153999 | hypo |
| cg22272803 | chr7 | 155989369 | OpenSea |  |  | 6.53E-08 | 0.00162626 | HYPER |
| cg21106962 | chr10 | 62150977 | OpenSea | TSS1500 | ANK3 | 7.39E-08 | 0.00178618 | hypo |
| cg04135540 | chr4 | 42296896 | OpenSea |  |  | 7.66E-08 | 0.00180053 | HYPER |
| cg13463245 | chr9 | 140042657 | Island | Body | GRIN1 | 8.09E-08 | 0.00184802 | hypo |
| cg07218625 | chr19 | 49956506 | Island | TSS1500 | PIH1D1 | 8.37E-08 | 0.00185972 | HYPER |
| cg08139234 | chr1 | 29240631 | N_Shore | 5'UTR | EPB41 | 8.91E-08 | 0.00192813 | HYPER |
| cg13464448 | chr11 | 130297513 | Island | 1stExon | ADAMTS8 | 1.05E-07 | 0.00218602 | HYPER |
| cg24342002 | chr20 | 25604715 | Island | TSS200 | NANP | 1.06E-07 | 0.00218602 | HYPER |
| cg02606505 | chr3 | 42689703 | OpenSea | 3'UTR | NKTR | 1.46E-07 | 0.00285607 | hypo |
| cg16646539 | chr4 | 88141915 | Island | TSS1500 | KLHL8 | 1.44E-07 | 0.00285607 | HYPER |
| cg13745593 | chr4 | 135248186 | OpenSea |  |  | 1.71E-07 | 0.0032684 | hypo |
| cg07669814 | chr10 | 52388333 | S_Shelf |  |  | 1.82E-07 | 0.00340179 | hypo |
| cg10413136 | chr4 | 8207119 | OpenSea | Body | SH3TC1 | 1.95E-07 | 0.00355929 | hypo |
| cg12471811 | chr10 | 49813371 | S_Shore | TSS1500 | ARHGAP22 | 2.30E-07 | 0.00408157 | HYPER |
| cg25931868 | chr18 | 32531847 | OpenSea |  |  | 2.33E-07 | 0.00408157 | hypo |
| cg16540981 | chr1 | 112016558 | OpenSea | 1stExon | C1orf162 | 2.39E-07 | 0.00408801 | hypo |
| cg09892203 | chr17 | 64961140 | Island | 1stExon | CACNG4 | 2.47E-07 | 0.00413848 | HYPER |
| cg08357850 | chr16 | 74734885 | S_Shore | TSS200 | MLKL | 2.81E-07 | 0.00462339 | HYPER |
| cg19224713 | chr8 | 119634283 | Island | TSS200 | SAMD12 | 2.87E-07 | 0.00462882 | HYPER |
| cg08446110 | chr4 | 68416686 | OpenSea |  |  | 2.95E-07 | 0.00467075 | hypo |
| cg08106148 | chr6 | 130339617 | N_Shore | TSS200 | L3MBTL3 | 3.08E-07 | 0.0047819 | HYPER |
| cg08956303 | chr11 | 45967551 | OpenSea | ExonBnd | PHF21A | 3.50E-07 | 0.00518306 | hypo |
| cg10420089 | chr6 | 151187322 | Island | TSS200 | MTHFD1L | 3.57E-07 | 0.00518306 | hypo |
| cg12449155 | chr14 | 70368487 | OpenSea | Body | SMOC1 | 3.51E-07 | 0.00518306 | HYPER |
| cg17619755 | chr6 | 31760629 | N_Shelf | Body | VARS | 3.59E-07 | 0.00518306 | HYPER |
| cg00353953 | chr12 | 6799541 | S_Shore | TSS1500 | ZNF384 | 3.96E-07 | 0.00562035 | HYPER |
| cg27128489 | chr16 | 46865867 | S_Shore | TSS1500 | C16orf87 | 4.19E-07 | 0.00584333 | hypo |
| cg19612173 | chr12 | 115121567 | Island | 1stExon | TBX3 | 4.92E-07 | 0.00674368 | HYPER |
| cg10160567 | chr3 | 69788520 | Island | TSS200 | MITF | 5.32E-07 | 0.00695801 | HYPER |
| cg10767216 | chr7 | 130130187 | N_Shore | Body | MESTIT1 | 5.33E-07 | 0.00695801 | hypo |
| cg10808367 | chr10 | 134451193 | OpenSea | Body | INPP5A | 5.19E-07 | 0.00695801 | hypo |
| cg09306584 | chr19 | 21579833 | OpenSea | TSS200 | ZNF493 | 5.56E-07 | 0.00713914 | HYPER |
| cg21303179 | chr12 | 132300008 | OpenSea |  |  | 5.68E-07 | 0.00718229 | hypo |
| cg08433912 | chr1 | 106765205 | OpenSea |  |  | 6.03E-07 | 0.00751134 | HYPER |
| cg06233497 | chr11 | 120200253 | S_Shelf | Body | TMEM136 | 6.70E-07 | 0.00822236 | hypo |
| cg26837844 | chr16 | 28969861 | OpenSea | TSS200 | MIR4517 | 6.82E-07 | 0.00824943 | hypo |
| cg04054036 | chr14 | 65273407 | OpenSea | Body | SPTB | 7.42E-07 | 0.00872387 | HYPER |
| cg26012716 | chr11 | 86748913 | Island | 1stExon | TMEM135 | 7.43E-07 | 0.00872387 | HYPER |
| cg15836199 | chr1 | 2302005 | N_Shelf | Body | MORN1 | 8.53E-07 | 0.00947975 | hypo |
| cg20884984 | chr11 | 119292770 | Island | 5'UTR | THY1 | 8.43E-07 | 0.00947975 | HYPER |
| cg23314508 | chr5 | 97134253 | OpenSea |  |  | 8.37E-07 | 0.00947975 | hypo |
| cg23907053 | chr12 | 70215816 | OpenSea | 3'UTR | RAB3IP | 8.27E-07 | 0.00947975 | HYPER |
| cg24232444 | chr13 | 99545448 | OpenSea | Body | DOCK9 | 8.65E-07 | 0.00948525 | hypo |
| cg23395795 | chr3 | 191863246 | OpenSea | Body | FGF12 | 8.99E-07 | 0.00972681 | hypo |
| cg11385067 | chr4 | 68751543 | OpenSea |  |  | 9.25E-07 | 0.00988248 | HYPER |
| cg03870044 | chr5 | 125966247 | OpenSea | TSS1500 | C5orf48 | 1.03E-06 | 0.01080954 | hypo |
| cg00275967 | chr13 | 106716455 | OpenSea |  |  | 1.08E-06 | 0.0112367 | HYPER |
| cg03510444 | chr2 | 26261517 | S_Shelf | Body | RAB10 | 1.18E-06 | 0.01215778 | hypo |
| cg06528466 | chr15 | 91010624 | OpenSea | Body | IQGAP1 | 1.21E-06 | 0.0122776 | hypo |
| cg03529803 | chr13 | 75794064 | OpenSea |  |  | 1.32E-06 | 0.0132554 | HYPER |
| cg09509433 | chr4 | 119691545 | OpenSea | Body | SEC24D | 1.44E-06 | 0.01428094 | hypo |
| cg24129484 | chr9 | 85681857 | S_Shelf |  |  | 1.47E-06 | 0.01434297 | HYPER |
| cg26124780 | chr2 | 86398889 | OpenSea | Body | IMMT | 1.50E-06 | 0.01439736 | hypo |
| ch.9.1865022F | chr9 | 120533485 | OpenSea |  |  | 1.51E-06 | 0.01439736 | HYPER |
| cg26769186 | chr3 | 196010417 | N_Shelf | 5'UTR | PCYT1A | 1.53E-06 | 0.01442106 | hypo |
| cg16486564 | chr16 | 46513219 | OpenSea | Body | ANKRD26P1 | 1.70E-06 | 0.01592833 | HYPER |
| cg10153212 | chr19 | 11370382 | N_Shelf | Body | DOCK6 | 1.88E-06 | 0.01720785 | HYPER |
| cg15900159 | chr13 | 24421418 | OpenSea | Body | MIPEP | 1.88E-06 | 0.01720785 | hypo |
| cg25743719 | chr2 | 10443138 | Island | 5'UTR | HPCAL1 | 1.90E-06 | 0.01720785 | HYPER |
| cg07116596 | chr5 | 131926919 | OpenSea | ExonBnd | RAD50 | 1.99E-06 | 0.01745038 | hypo |
| cg07515071 | chr12 | 42737622 | OpenSea | Body | PPHLN1 | 1.99E-06 | 0.01745038 | HYPER |
| cg10958924 | chr15 | 37391438 | N_Shore | 5'UTR | MEIS2 | 1.97E-06 | 0.01745038 | hypo |
| cg06873652 | chr11 | 82961275 | OpenSea | Body | ANKRD42 | 2.12E-06 | 0.01815948 | HYPER |
| cg13118761 | chr14 | 89293608 | S_Shelf | Body | TTC8 | 2.10E-06 | 0.01815948 | hypo |
| cg18675616 | chr4 | 1962842 | OpenSea | Body | WHSC1 | 2.18E-06 | 0.01846299 | hypo |
| cg03443887 | chr11 | 126138300 | N_Shore | TSS1500 | FOXRED1 | 2.23E-06 | 0.01863519 | HYPER |
| cg09175843 | chr2 | 70313417 | Island | TSS1500 | PCBP1 | 2.24E-06 | 0.01863519 | HYPER |
| cg18361081 | chr17 | 46385350 | OpenSea | Body | SKAP1 | 2.31E-06 | 0.01902745 | HYPER |
| cg25551551 | chr6 | 111808596 | S_Shelf |  |  | 2.38E-06 | 0.01935321 | hypo |
| cg03968783 | chr13 | 37399523 | OpenSea | Body | RFXAP | 2.57E-06 | 0.02035568 | hypo |
| cg11611793 | chr8 | 30003498 | OpenSea | TSS1500 | MBOAT4 | 2.57E-06 | 0.02035568 | hypo |
| cg14438434 | chr11 | 134038489 | OpenSea | ExonBnd | NCAPD3 | 2.53E-06 | 0.02035568 | hypo |
| cg09889646 | chr2 | 48692092 | OpenSea | ExonBnd | PPP1R21 | 2.64E-06 | 0.02069767 | hypo |
| cg06221470 | chr7 | 2353869 | Island | Body | SNX8 | 2.78E-06 | 0.02159913 | HYPER |
| cg22186263 | chr7 | 137570950 | OpenSea | Body | CREB3L2 | 2.87E-06 | 0.02207282 | hypo |
| cg23970630 | chr18 | 2541371 | OpenSea | Body | METTL4 | 2.95E-06 | 0.02242322 | hypo |
| cg15071899 | chr7 | 156228452 | OpenSea |  |  | 3.03E-06 | 0.02266069 | HYPER |
| cg22317649 | chr14 | 36225113 | OpenSea | Body | RALGAPA1P | 3.03E-06 | 0.02266069 | hypo |
| cg14327541 | chr9 | 86597173 | S_Shore | 5'UTR | RMI1 | 3.08E-06 | 0.02282547 | hypo |
| ch.2.1580620R | chr2 | 69966976 | N_Shore |  |  | 3.11E-06 | 0.02283434 | HYPER |
| cg08655465 | chr11 | 13894916 | OpenSea |  |  | 3.29E-06 | 0.02373573 | hypo |
| cg13858974 | chr9 | 86149293 | N_Shelf | Body | FRMD3 | 3.27E-06 | 0.02373573 | HYPER |
| cg16294016 | chr4 | 190862268 | Island | Body | FRG1 | 3.45E-06 | 0.0247013 | HYPER |
| cg03179941 | chr11 | 35768455 | OpenSea | Body | TRIM44 | 3.61E-06 | 0.02498652 | hypo |
| cg05687194 | chr16 | 84178061 | Island | 5'UTR | HSDL1 | 3.62E-06 | 0.02498652 | HYPER |
| cg14375050 | chr4 | 108848533 | N_Shelf |  |  | 3.59E-06 | 0.02498652 | hypo |
| cg24966735 | chr13 | 74708143 | Island | TSS200 | KLF12 | 3.57E-06 | 0.02498652 | HYPER |
| cg02416873 | chr21 | 30502731 | OpenSea | TSS200 | MAP3K7CL | 3.68E-06 | 0.02511162 | HYPER |
| cg24336123 | chr20 | 34286939 | Island | TSS1500 | ROMO1 | 3.73E-06 | 0.02511162 | HYPER |
| cg26881603 | chr22 | 21213153 | Island | TSS200 | SNAP29 | 3.70E-06 | 0.02511162 | HYPER |
| cg18659719 | chr7 | 88258394 | OpenSea |  |  | 3.78E-06 | 0.02525428 | HYPER |
| cg01028360 | chr8 | 12869510 | OpenSea | Body | C8orf79 | 3.96E-06 | 0.02625589 | HYPER |
| cg06428121 | chr12 | 63497523 | OpenSea |  |  | 4.01E-06 | 0.02640595 | HYPER |
| cg06865629 | chr18 | 19409164 | OpenSea | TSS200 | MIR1-2 | 4.18E-06 | 0.02725252 | hypo |
| cg10321252 | chr15 | 45708887 | OpenSea | Body | SPATA5L1 | 4.23E-06 | 0.02740957 | hypo |
| cg14732655 | chr18 | 71186297 | OpenSea |  |  | 4.35E-06 | 0.02751056 | HYPER |
| cg17350441 | chr10 | 88389642 | N_Shelf |  |  | 4.29E-06 | 0.02751056 | hypo |
| cg24641993 | chr5 | 150537046 | Island | 5'UTR | ANXA6 | 4.32E-06 | 0.02751056 | HYPER |
| cg25303774 | chr6 | 121902246 | OpenSea |  |  | 4.42E-06 | 0.02775246 | hypo |
| cg14789080 | chr13 | 70367962 | OpenSea | Body | KLHL1 | 4.47E-06 | 0.02782825 | HYPER |
| cg21169285 | chr5 | 179707665 | OpenSea | 5'UTR | MAPK9 | 4.51E-06 | 0.02788999 | hypo |
| cg04165709 | chr19 | 53117949 | OpenSea | 5'UTR | ZNF83 | 4.58E-06 | 0.0280756 | hypo |
| cg04252889 | chr22 | 32756957 | OpenSea | 3'UTR | RFPL3 | 4.66E-06 | 0.02817607 | HYPER |
| cg10679265 | chr5 | 33460568 | OpenSea | Body | TARS | 4.64E-06 | 0.02817607 | hypo |
| cg03329533 | chr13 | 47117138 | OpenSea |  |  | 4.75E-06 | 0.02841809 | hypo |
| cg05989363 | chr11 | 9418531 | OpenSea | Body | IPO7 | 4.77E-06 | 0.02841809 | HYPER |
| cg01958738 | chr1 | 243556057 | OpenSea | Body | SDCCAG8 | 4.84E-06 | 0.02842507 | hypo |
| cg09010938 | chr4 | 84678805 | OpenSea |  |  | 4.82E-06 | 0.02842507 | hypo |
| cg25342510 | chr14 | 35183231 | N_Shore | Body | CFL2 | 4.94E-06 | 0.02881998 | HYPER |
| cg10635005 | chr6 | 139629426 | OpenSea |  |  | 5.12E-06 | 0.0296743 | hypo |
| cg11292807 | chr13 | 99922428 | OpenSea | Body | UBAC2 | 5.49E-06 | 0.03103037 | hypo |
| cg19508435 | chr11 | 28131980 | Island | 5'UTR | METT5D1 | 5.51E-06 | 0.03103037 | HYPER |
| cg23361197 | chr12 | 95461590 | OpenSea | 5'UTR | NR2C1 | 5.46E-06 | 0.03103037 | hypo |
| cg26408837 | chr17 | 77334776 | OpenSea | 5'UTR | RBFOX3 | 5.45E-06 | 0.03103037 | HYPER |
| cg15672986 | chr3 | 175465989 | OpenSea | Body | NAALADL2 | 5.56E-06 | 0.03110128 | hypo |
| cg03511402 | chr16 | 30583225 | Island | 5'UTR | ZNF688 | 5.73E-06 | 0.03162763 | HYPER |
| cg06810293 | chr6 | 34660294 | N_Shelf | Body | C6orf106 | 5.70E-06 | 0.03162763 | hypo |
| cg09376537 | chr17 | 48638103 | Island | TSS1500 | CACNA1G | 5.80E-06 | 0.03178906 | HYPER |
| cg15438999 | chr2 | 97538522 | S_Shelf |  |  | 5.84E-06 | 0.03181666 | HYPER |
| cg18989243 | chr1 | 115259527 | OpenSea | TSS200 | NRAS | 5.91E-06 | 0.0319674 | HYPER |
| cg12368809 | chr9 | 108337268 | OpenSea | 5'UTR | FKTN | 6.01E-06 | 0.03227594 | hypo |
| cg00580291 | chr13 | 21606284 | OpenSea | Body | LATS2 | 6.30E-06 | 0.03320495 | hypo |
| cg02833116 | chr7 | 108210556 | Island | TSS1500 | THAP5 | 6.38E-06 | 0.03320495 | HYPER |
| cg12195054 | chr13 | 110441343 | S_Shore |  |  | 6.33E-06 | 0.03320495 | hypo |
| cg19250045 | chr8 | 122543761 | OpenSea |  |  | 6.26E-06 | 0.03320495 | HYPER |
| cg19533231 | chr18 | 54814390 | OpenSea |  |  | 6.38E-06 | 0.03320495 | hypo |
| cg25283172 | chr2 | 29038696 | OpenSea | 5'UTR | SPDYA | 6.48E-06 | 0.03352683 | hypo |
| cg02335700 | chr12 | 110282809 | N_Shore |  |  | 6.64E-06 | 0.03389783 | HYPER |
| cg09925124 | chr2 | 8868639 | OpenSea |  |  | 6.65E-06 | 0.03389783 | hypo |
| cg22343476 | chr1 | 225845563 | S_Shelf |  |  | 6.68E-06 | 0.03389783 | HYPER |
| cg09688285 | chr10 | 111682808 | Island | Body | XPNPEP1 | 6.76E-06 | 0.03408254 | HYPER |
| cg25247636 | chr19 | 6940227 | OpenSea | 3'UTR | EMR1 | 7.33E-06 | 0.03677416 | HYPER |
| cg02317534 | chr7 | 22705333 | Island |  |  | 7.57E-06 | 0.03707245 | hypo |
| cg13690593 | chr6 | 7113051 | S_Shelf | 5'UTR | RREB1 | 7.51E-06 | 0.03707245 | hypo |
| cg19630681 | chr3 | 64429607 | N_Shore |  |  | 7.57E-06 | 0.03707245 | HYPER |
| cg21314480 | chr2 | 75427945 | Island | TSS1500 | TACR1 | 7.51E-06 | 0.03707245 | HYPER |
| cg23910098 | chr11 | 3187534 | S_Shore | TSS1500 | OSBPL5 | 7.72E-06 | 0.03753468 | hypo |
| ch.9.2473665R | chr9 | 139311799 | N_Shore | Body | PMPCA | 7.76E-06 | 0.03753468 | HYPER |
| cg24305576 | chr14 | 77838396 | OpenSea | Body | TMED8 | 7.83E-06 | 0.03765199 | hypo |
| cg08313132 | chrX | 15873151 | Island | TSS200 | AP1S2 | 7.89E-06 | 0.03772298 | HYPER |
| cg07365274 | chr3 | 114866242 | Island | TSS200 | ZBTB20 | 8.12E-06 | 0.03838786 | HYPER |
| cg13361488 | chr11 | 126114136 | OpenSea | Body | FAM118B | 8.12E-06 | 0.03838786 | HYPER |
| ch.20.6400133F | chr20 | 6452133 | OpenSea |  |  | 8.18E-06 | 0.03841652 | HYPER |
| cg14286594 | chr4 | 140245762 | OpenSea | Body | NAA15 | 8.54E-06 | 0.03990232 | hypo |
| cg23906144 | chr17 | 1945509 | Island | 1stExon | OVCA2 | 9.07E-06 | 0.04211833 | HYPER |
| cg11947509 | chr2 | 202898349 | Island | TSS1500 | FZD7 | 9.33E-06 | 0.04311849 | hypo |
| cg27415969 | chr3 | 186500848 | Island | TSS1500 | EIF4A2 | 9.52E-06 | 0.04372153 | HYPER |
| cg21186771 | chr2 | 73287456 | OpenSea | Body | SFXN5 | 9.96E-06 | 0.04551897 | hypo |
| cg19260592 | chr1 | 98872441 | OpenSea |  |  | 1.01E-05 | 0.04580723 | hypo |
| cg00222995 | chr6 | 33421704 | N_Shore | TSS1500 | ZBTB9 | 1.03E-05 | 0.04617783 | HYPER |
| cg01071385 | chr6 | 109062212 | OpenSea |  |  | 1.03E-05 | 0.04617783 | hypo |
| cg02348711 | chr17 | 41623836 | Island | TSS1500 | ETV4 | 1.04E-05 | 0.04663932 | HYPER |
| cg04217534 | chr11 | 82354913 | OpenSea |  |  | 1.06E-05 | 0.04700364 | HYPER |
| cg00276792 | chr16 | 3208796 | Island |  |  | 1.08E-05 | 0.04761012 | hypo |
| cg06692315 | chr2 | 70417854 | Island | 5'UTR | C2orf42 | 1.10E-05 | 0.04807966 | HYPER |
| cg10978468 | chr17 | 57732531 | OpenSea | Body | CLTC | 1.11E-05 | 0.04807966 | hypo |
| cg13174599 | chr7 | 131547013 | OpenSea |  |  | 1.10E-05 | 0.04807966 | hypo |
| cg22235926 | chr5 | 56131369 | OpenSea | Body | MAP3K1 | 1.11E-05 | 0.04807966 | hypo |
| cg06500727 | chr2 | 1417164 | OpenSea | TSS1500 | TPO | 1.12E-05 | 0.04833485 | HYPER |
| cg04774620 | chr3 | 78765904 | OpenSea | Body | ROBO1 | 1.14E-05 | 0.04888149 | hypo |
| cg08344447 | chr9 | 112945357 | OpenSea |  |  | 1.15E-05 | 0.04897176 | HYPER |
| cg07202461 | chr18 | 19406765 | OpenSea | Body | MIB1 | 1.18E-05 | 0.04993176 | hypo |

| **Name** | **Chr** | **Position** | **CpG Island** | **Region** | **Related gene** | **P.value** | **FDR** | **Methyl** |
| --- | --- | --- | --- | --- | --- | --- | --- | --- |
| **IGF-1** | | | | | | | | |
| cg07926598 | chr7 | 4175200 | OpenSea | Body | SDK1 | 4.74E-12 | 3.90E-06 | HYPER |
| cg20227471 | chr2 | 25065550 | OpenSea | Body | ADCY3 | 3.15E-11 | 5.18E-06 | hypo |
| cg07587588 | chr8 | 81083356 | Island | Body | TPD52 | 2.61E-11 | 5.18E-06 | HYPER |
| cg22855933 | chr2 | 157179512 | S_Shelf |  |  | 2.57E-11 | 5.18E-06 | hypo |
| cg18530551 | chr5 | 172670879 | Island |  |  | 2.16E-11 | 5.18E-06 | hypo |
| cg25755428 | chr19 | 13875111 | Island | TSS1500 | MRI1 | 6.18E-11 | 8.46E-06 | HYPER |
| cg26951364 | chrX | 133306382 | Island |  |  | 7.74E-11 | 9.10E-06 | HYPER |
| cg17412258 | chr14 | 101193017 | Island | TSS1500 | DLK1 | 1.03E-10 | 1.06E-05 | hypo |
| cg23536255 | chr6 | 31276105 | N_Shore |  |  | 1.61E-10 | 1.47E-05 | HYPER |
| cg12664940 | chr5 | 140430875 | OpenSea | TSS200 | PCDHB1 | 3.05E-10 | 2.50E-05 | hypo |
| cg15578091 | chr1 | 236908064 | OpenSea | Body | ACTN2 | 3.56E-10 | 2.66E-05 | hypo |
| cg19591512 | chr3 | 134514284 | Island | 5'UTR | EPHB1 | 9.80E-10 | 6.20E-05 | HYPER |
| cg16967640 | chr1 | 165205453 | Island | Body | LMX1A | 9.50E-10 | 6.20E-05 | hypo |
| cg17301223 | chr8 | 145106438 | Island | Body | OPLAH | 2.33E-09 | 0.000137 | HYPER |
| cg23658477 | chr12 | 22094371 | Island |  |  | 6.66E-09 | 0.00036516 | HYPER |
| cg05183229 | chr10 | 25012543 | Island | 1stExon | ARHGAP21 | 9.68E-09 | 0.00044287 | hypo |
| cg15628498 | chr15 | 53082902 | Island | TSS1500 | ONECUT1 | 9.70E-09 | 0.00044287 | HYPER |
| cg17504203 | chr16 | 89894813 | Island | TSS200 | SPIRE2 | 9.38E-09 | 0.00044287 | HYPER |
| cg07202461 | chr18 | 19406765 | OpenSea | Body | MIB1 | 1.38E-08 | 0.00054164 | hypo |
| cg04135540 | chr4 | 42296896 | OpenSea |  |  | 1.35E-08 | 0.00054164 | HYPER |
| cg04182363 | chr8 | 81316879 | OpenSea |  |  | 1.37E-08 | 0.00054164 | hypo |
| cg17466535 | chr14 | 20929606 | Island | 5'UTR | TMEM55B | 1.49E-08 | 0.00055613 | hypo |
| cg25472353 | chr7 | 37489005 | S_Shore | TSS1500 | ELMO1 | 1.62E-08 | 0.00056095 | hypo |
| cg07627556 | chr11 | 115530590 | S_Shore |  |  | 1.64E-08 | 0.00056095 | HYPER |
| cg19250045 | chr8 | 122543761 | OpenSea |  |  | 2.56E-08 | 0.0008414 | HYPER |
| cg00876541 | chr19 | 10515578 | Island | TSS1500 | MIR1181 | 2.88E-08 | 0.00091177 | HYPER |
| cg08139234 | chr1 | 29240631 | N_Shore | 5'UTR | EPB41 | 3.59E-08 | 0.00109422 | HYPER |
| cg25497529 | chr19 | 18901364 | Island | Body | COMP | 4.47E-08 | 0.00126634 | hypo |
| cg11497372 | chr6 | 27513092 | OpenSea |  |  | 4.44E-08 | 0.00126634 | HYPER |
| cg15157241 | chr21 | 44396204 | S_Shore | 5'UTR | PKNOX1 | 5.61E-08 | 0.00153854 | hypo |
| cg09521108 | chr4 | 74735149 | Island | 5'UTR | CXCL1 | 6.06E-08 | 0.00155604 | HYPER |
| cg07414687 | chr1 | 203240341 | N_Shelf |  |  | 6.03E-08 | 0.00155604 | HYPER |
| cg11859154 | chr5 | 33299505 | OpenSea |  |  | 7.63E-08 | 0.0019004 | hypo |
| cg02664390 | chr1 | 179911884 | OpenSea |  |  | 7.86E-08 | 0.00190168 | hypo |
| cg09892203 | chr17 | 64961140 | Island | 1stExon | CACNG4 | 8.65E-08 | 0.00203132 | HYPER |
| cg16487794 | chr1 | 64936656 | S_Shore | Body | CACHD1 | 9.85E-08 | 0.00224869 | HYPER |
| cg13463245 | chr9 | 140042657 | Island | Body | GRIN1 | 1.06E-07 | 0.00236179 | hypo |
| cg14732655 | chr18 | 71186297 | OpenSea |  |  | 1.09E-07 | 0.00236179 | HYPER |
| cg13464448 | chr11 | 130297513 | Island | 1stExon | ADAMTS8 | 1.19E-07 | 0.00251129 | HYPER |
| cg16540981 | chr1 | 112016558 | OpenSea | 1stExon | C1orf162 | 1.28E-07 | 0.00256578 | hypo |
| cg10030268 | chr2 | 234465509 | OpenSea | ExonBnd | USP40 | 1.26E-07 | 0.00256578 | hypo |
| cg05578055 | chr8 | 16859045 | Island | Body | FGF20 | 1.37E-07 | 0.00267797 | HYPER |
| cg02606505 | chr3 | 42689703 | OpenSea | 3'UTR | NKTR | 1.47E-07 | 0.00280666 | hypo |
| cg17525025 | chr16 | 24740470 | N_Shore | TSS1500 | TNRC6A | 1.66E-07 | 0.00309284 | hypo |
| cg26499542 | chr5 | 42565472 | OpenSea | TSS200 | GHR | 1.87E-07 | 0.00341528 | HYPER |
| cg08344447 | chr9 | 112945357 | OpenSea |  |  | 1.95E-07 | 0.00348816 | HYPER |
| cg24342002 | chr20 | 25604715 | Island | TSS200 | NANP | 2.12E-07 | 0.00363119 | HYPER |
| cg10413136 | chr4 | 8207119 | OpenSea | Body | SH3TC1 | 2.12E-07 | 0.00363119 | hypo |
| cg08956303 | chr11 | 45967551 | OpenSea | ExonBnd | PHF21A | 2.22E-07 | 0.00371761 | hypo |
| cg10652054 | chr13 | 43649126 | OpenSea | Body | DNAJC15 | 2.61E-07 | 0.00429492 | hypo |
| cg13720284 | chr8 | 6940591 | OpenSea |  |  | 2.78E-07 | 0.00448409 | hypo |
| cg26752263 | chr6 | 53409853 | Island | TSS200 | GCLC | 3.04E-07 | 0.00480738 | HYPER |
| cg16486564 | chr16 | 46513219 | OpenSea | Body | ANKRD26P1 | 3.24E-07 | 0.00502778 | HYPER |
| cg08106148 | chr6 | 130339617 | N_Shore | TSS200 | L3MBTL3 | 3.46E-07 | 0.0052723 | HYPER |
| cg03336120 | chr13 | 69561352 | S_Shelf |  |  | 3.55E-07 | 0.00531403 | hypo |
| cg06865629 | chr18 | 19409164 | OpenSea | TSS200 | MIR1-2 | 3.73E-07 | 0.00539138 | hypo |
| cg15859867 | chr12 | 57735981 | OpenSea |  |  | 3.74E-07 | 0.00539138 | hypo |
| cg04487907 | chr19 | 9326161 | OpenSea | TSS1500 | OR7D4 | 3.81E-07 | 0.00539602 | HYPER |
| cg17502532 | chr9 | 101558996 | Island | TSS200 | ANKS6 | 4.12E-07 | 0.00555097 | HYPER |
| cg15836199 | chr1 | 2302005 | N_Shelf | Body | MORN1 | 4.21E-07 | 0.00555097 | hypo |
| cg22695998 | chr12 | 56214735 | S_Shelf | 3'UTR | ORMDL2 | 4.18E-07 | 0.00555097 | hypo |
| cg23906144 | chr17 | 1945509 | Island | 1stExon | OVCA2 | 4.25E-07 | 0.00555097 | HYPER |
| cg25303774 | chr6 | 121902246 | OpenSea |  |  | 4.16E-07 | 0.00555097 | hypo |
| cg08357850 | chr16 | 74734885 | S_Shore | TSS200 | MLKL | 4.32E-07 | 0.00555574 | HYPER |
| cg10153212 | chr19 | 11370382 | N_Shelf | Body | DOCK6 | 4.40E-07 | 0.00556317 | HYPER |
| cg22343476 | chr1 | 225845563 | S_Shelf |  |  | 4.77E-07 | 0.00594839 | HYPER |
| cg16670476 | chr14 | 74600830 | OpenSea | Body | LIN52 | 5.32E-07 | 0.00643564 | hypo |
| cg27062293 | chr11 | 71759798 | OpenSea | Body | NUMA1 | 5.26E-07 | 0.00643564 | hypo |
| cg00580291 | chr13 | 21606284 | OpenSea | Body | LATS2 | 5.56E-07 | 0.00662624 | hypo |
| cg17619755 | chr6 | 31760629 | N_Shelf | Body | VARS | 5.73E-07 | 0.00673143 | HYPER |
| cg08927962 | chr9 | 3267967 | OpenSea | Body | RFX3 | 6.20E-07 | 0.00698248 | HYPER |
| cg01482376 | chr11 | 12913606 | OpenSea | Body | TEAD1 | 6.28E-07 | 0.00698248 | hypo |
| cg07201934 | chr7 | 112410323 | OpenSea | Body | TMEM168 | 6.28E-07 | 0.00698248 | hypo |
| cg14375896 | chr1 | 25785707 | OpenSea | Body | TMEM57 | 6.16E-07 | 0.00698248 | hypo |
| cg10767216 | chr7 | 130130187 | N_Shore | Body | MESTIT1 | 6.75E-07 | 0.00739832 | hypo |
| cg23372016 | chr6 | 80892124 | OpenSea | Body | BCKDHB | 7.19E-07 | 0.00770489 | HYPER |
| cg21267439 | chr6 | 116565566 | OpenSea | 3'UTR | NT5DC1 | 7.22E-07 | 0.00770489 | hypo |
| cg06810293 | chr6 | 34660294 | N_Shelf | Body | C6orf106 | 7.59E-07 | 0.0080007 | hypo |
| cg00682427 | chr6 | 83882969 | OpenSea | Body | PGM3 | 8.05E-07 | 0.00837591 | hypo |
| cg08864240 | chr6 | 39895717 | OpenSea | Body | MOCS1 | 8.55E-07 | 0.00878818 | hypo |
| cg02951237 | chr13 | 45545983 | OpenSea | Body | NUFIP1 | 8.88E-07 | 0.0090099 | hypo |
| cg16646539 | chr4 | 88141915 | Island | TSS1500 | KLHL8 | 9.22E-07 | 0.0092415 | HYPER |
| cg02723533 | chr11 | 69468991 | Island | 3'UTR | CCND1 | 1.00E-06 | 0.00991106 | hypo |
| cg11031647 | chrX | 110654011 | OpenSea | 1stExon | DCX | 1.02E-06 | 0.01000477 | HYPER |
| cg18675616 | chr4 | 1962842 | OpenSea | Body | WHSC1 | 1.13E-06 | 0.01091049 | hypo |
| cg26200347 | chr10 | 15254612 | Island | 3'UTR | FAM171A1 | 1.17E-06 | 0.01103416 | hypo |
| cg15123849 | chr6 | 15364953 | OpenSea | Body | JARID2 | 1.16E-06 | 0.01103416 | hypo |
| cg23692214 | chr10 | 126631393 | OpenSea | 1stExon | ZRANB1 | 1.18E-06 | 0.01103461 | hypo |
| cg06047551 | chr14 | 55834080 | OpenSea | 3'UTR | ATG14 | 1.20E-06 | 0.01106432 | hypo |
| cg15536804 | chr12 | 26647178 | OpenSea | Body | ITPR2 | 1.24E-06 | 0.01128288 | hypo |
| cg13745593 | chr4 | 135248186 | OpenSea |  |  | 1.25E-06 | 0.01128288 | hypo |
| cg11839944 | chr11 | 17409644 | Island | 1stExon | KCNJ11 | 1.33E-06 | 0.01181689 | hypo |
| cg00720106 | chr15 | 102189106 | N_Shelf | Body | TM2D3 | 1.34E-06 | 0.01181689 | hypo |
| cg22272803 | chr7 | 155989369 | OpenSea |  |  | 1.37E-06 | 0.01198413 | HYPER |
| cg03962773 | chr8 | 85578293 | OpenSea | Body | RALYL | 1.41E-06 | 0.01219441 | HYPER |
| cg25283172 | chr2 | 29038696 | OpenSea | 5'UTR | SPDYA | 1.45E-06 | 0.01241515 | hypo |
| cg14819729 | chrX | 19050646 | OpenSea | Body | ADGRG2 | 1.54E-06 | 0.01304366 | hypo |
| cg19612173 | chr12 | 115121567 | Island | 1stExon | TBX3 | 1.61E-06 | 0.01354752 | HYPER |
| cg11035992 | chr17 | 7657473 | OpenSea | TSS200 | RPL29P2 | 1.63E-06 | 0.01356819 | hypo |
| cg21106962 | chr10 | 62150977 | OpenSea | TSS1500 | ANK3 | 1.71E-06 | 0.01398165 | hypo |
| cg03511402 | chr16 | 30583225 | Island | 5'UTR | ZNF688 | 1.72E-06 | 0.01398165 | HYPER |
| cg16290059 | chr3 | 139082156 | OpenSea | Body | COPB2 | 1.85E-06 | 0.0146423 | hypo |
| cg06221470 | chr7 | 2353869 | Island | Body | SNX8 | 1.84E-06 | 0.0146423 | HYPER |
| cg17500817 | chr12 | 44467155 | OpenSea | 5'UTR | TMEM117 | 1.85E-06 | 0.0146423 | HYPER |
| cg00275967 | chr13 | 106716455 | OpenSea |  |  | 1.87E-06 | 0.01467394 | HYPER |
| cg04054036 | chr14 | 65273407 | OpenSea | Body | SPTB | 1.94E-06 | 0.0150274 | HYPER |
| cg02251850 | chr17 | 78851503 | S_Shore | Body | RPTOR | 2.12E-06 | 0.01627718 | hypo |
| cg22727109 | chr12 | 32112813 | Island | 5'UTR | C12orf35 | 2.16E-06 | 0.01643616 | hypo |
| cg09954543 | chr5 | 176853633 | Island | TSS200 | GRK6 | 2.20E-06 | 0.01657354 | HYPER |
| cg24688343 | chr17 | 61229244 | OpenSea | Body | TANC2 | 2.26E-06 | 0.01685743 | hypo |
| cg09940936 | chr3 | 149589902 | OpenSea | Body | RNF13 | 2.35E-06 | 0.01731473 | hypo |
| cg21186771 | chr2 | 73287456 | OpenSea | Body | SFXN5 | 2.36E-06 | 0.01731473 | hypo |
| cg08655465 | chr11 | 13894916 | OpenSea |  |  | 2.41E-06 | 0.01753859 | hypo |
| cg09889646 | chr2 | 48692092 | OpenSea | ExonBnd | PPP1R21 | 2.48E-06 | 0.01788476 | hypo |
| cg13864354 | chr6 | 136206116 | OpenSea | Body | PDE7B | 2.51E-06 | 0.01791487 | hypo |
| cg15071899 | chr7 | 156228452 | OpenSea |  |  | 2.61E-06 | 0.01846726 | HYPER |
| cg15954386 | chr4 | 169240021 | S_Shore | TSS200 | DDX60 | 2.67E-06 | 0.01860365 | HYPER |
| cg11385067 | chr4 | 68751543 | OpenSea |  |  | 2.67E-06 | 0.01860365 | HYPER |
| cg26729242 | chr10 | 96980135 | OpenSea | Body | C10orf129 | 2.74E-06 | 0.01879605 | hypo |
| cg06139099 | chr10 | 134499505 | OpenSea | Body | INPP5A | 2.72E-06 | 0.01879605 | hypo |
| cg09021385 | chrX | 107552548 | OpenSea | Body | COL4A6 | 2.86E-06 | 0.01942604 | HYPER |
| cg11849422 | chr9 | 112542571 | Island | TSS200 | PALM2 | 2.93E-06 | 0.01977842 | HYPER |
| cg21795808 | chr4 | 76360190 | OpenSea |  |  | 3.00E-06 | 0.0200217 | HYPER |
| cg15917362 | chr14 | 30085301 | OpenSea | Body | PRKD1 | 3.03E-06 | 0.02006064 | hypo |
| cg05370471 | chr2 | 118594649 | S_Shore |  |  | 3.05E-06 | 0.02009422 | hypo |
| cg08616585 | chr16 | 68774986 | S_Shelf | Body | CDH1 | 3.14E-06 | 0.02031534 | hypo |
| cg05687194 | chr16 | 84178061 | Island | 5'UTR | HSDL1 | 3.13E-06 | 0.02031534 | HYPER |
| cg05252062 | chr7 | 101712308 | OpenSea | Body | CUX1 | 3.41E-06 | 0.02151123 | hypo |
| cg10808367 | chr10 | 134451193 | OpenSea | Body | INPP5A | 3.43E-06 | 0.02151123 | hypo |
| cg12277784 | chr8 | 107459734 | OpenSea | TSS1500 | OXR1 | 3.41E-06 | 0.02151123 | hypo |
| cg15681299 | chr1 | 167009265 | OpenSea |  |  | 3.39E-06 | 0.02151123 | HYPER |
| cg06576484 | chr7 | 92382242 | OpenSea | Body | CDK6 | 3.57E-06 | 0.02191155 | HYPER |
| ch.8.1073630F | chr8 | 48637817 | OpenSea | Body | KIAA0146 | 3.60E-06 | 0.02191155 | HYPER |
| cg23361197 | chr12 | 95461590 | OpenSea | 5'UTR | NR2C1 | 3.54E-06 | 0.02191155 | hypo |
| cg27201679 | chr10 | 1120794 | OpenSea | Body | WDR37 | 3.58E-06 | 0.02191155 | hypo |
| cg25098497 | chr12 | 13254403 | Island | Body | GSG1 | 3.68E-06 | 0.02227904 | HYPER |
| cg22355031 | chrX | 68060738 | S_Shore | 3'UTR | EFNB1 | 3.76E-06 | 0.02256446 | HYPER |
| cg02073954 | chr6 | 28193080 | Island | 5'UTR | ZNF193 | 3.83E-06 | 0.02279281 | HYPER |
| cg19217820 | chr11 | 101916810 | N_Shore | TSS1500 | C11orf70 | 3.86E-06 | 0.02286199 | hypo |
| cg21622630 | chr11 | 72158515 | OpenSea |  |  | 4.00E-06 | 0.02351665 | HYPER |
| cg14789080 | chr13 | 70367962 | OpenSea | Body | KLHL1 | 4.19E-06 | 0.02444022 | HYPER |
| cg20840002 | chr11 | 105924441 | OpenSea | Body | KBTBD3 | 4.54E-06 | 0.02608725 | hypo |
| cg11768148 | chr6 | 56910312 | N_Shore | TSS1500 | KIAA1586 | 4.51E-06 | 0.02608725 | hypo |
| cg24641993 | chr5 | 150537046 | Island | 5'UTR | ANXA6 | 4.72E-06 | 0.02692427 | HYPER |
| cg26576481 | chr12 | 76478884 | Island | TSS200 | NAP1L1 | 4.75E-06 | 0.02692427 | hypo |
| cg07334009 | chr1 | 170044710 | S_Shore | TSS1500 | KIFAP3 | 4.82E-06 | 0.02702277 | HYPER |
| cg10584042 | chr10 | 52383933 | Island | TSS200 | SGMS1 | 4.83E-06 | 0.02702277 | HYPER |
| cg12162100 | chr8 | 87529647 | S_Shelf | 5'UTR | CPNE3 | 5.25E-06 | 0.02909626 | hypo |
| cg14561362 | chr4 | 146461062 | OpenSea | Body | SMAD1 | 5.27E-06 | 0.02909626 | HYPER |
| cg08518329 | chr2 | 20101943 | Island | TSS200 | TTC32 | 5.33E-06 | 0.02922164 | HYPER |
| cg00276792 | chr16 | 3208796 | Island |  |  | 5.47E-06 | 0.02967856 | hypo |
| cg09725698 | chr5 | 39462344 | OpenSea |  |  | 5.49E-06 | 0.02967856 | hypo |
| cg08633697 | chrX | 39869105 | Island |  |  | 5.52E-06 | 0.0296788 | hypo |
| cg20704957 | chr5 | 87140414 | OpenSea |  |  | 5.56E-06 | 0.02970601 | HYPER |
| cg04689379 | chr19 | 46171262 | N_Shelf | TSS1500 | GIPR | 5.64E-06 | 0.02990372 | HYPER |
| cg05934755 | chr2 | 241498962 | N_Shore | TSS1500 | DUSP28 | 5.83E-06 | 0.03055389 | hypo |
| cg16341987 | chr2 | 228905135 | OpenSea | Body | SPHKAP | 5.83E-06 | 0.03055389 | hypo |
| cg13655566 | chr9 | 78536245 | OpenSea | Body | PCSK5 | 5.92E-06 | 0.03061781 | hypo |
| cg19225755 | chr6 | 132539768 | OpenSea |  |  | 5.92E-06 | 0.03061781 | HYPER |
| cg17851488 | chr3 | 45596195 | OpenSea |  |  | 5.98E-06 | 0.03071664 | hypo |
| cg17755518 | chr1 | 76077832 | N_Shelf | TSS1500 | SLC44A5 | 6.03E-06 | 0.03077168 | HYPER |
| cg19335003 | chr5 | 140185966 | N_Shore | TSS1500 | PCDHA4 | 6.25E-06 | 0.03171024 | HYPER |
| cg01175610 | chr12 | 12224246 | OpenSea | 5'UTR | BCL2L14 | 6.33E-06 | 0.03194184 | HYPER |
| cg08313132 | chrX | 15873151 | Island | TSS200 | AP1S2 | 6.45E-06 | 0.03235727 | HYPER |
| cg27457169 | chr17 | 38804285 | Island | TSS200 | SMARCE1 | 6.50E-06 | 0.03240343 | hypo |
| cg07982481 | chr10 | 75007255 | S_Shore | TSS1500 | DNAJC9 | 6.66E-06 | 0.0329916 | HYPER |
| cg09376537 | chr17 | 48638103 | Island | TSS1500 | CACNA1G | 7.43E-06 | 0.03595753 | HYPER |
| cg22768296 | chr6 | 4954316 | OpenSea | 3'UTR | CDYL | 7.33E-06 | 0.03595753 | hypo |
| cg00417576 | chr11 | 14535610 | OpenSea | Body | PSMA1 | 7.35E-06 | 0.03595753 | hypo |
| cg01872971 | chr4 | 82325484 | OpenSea |  |  | 7.42E-06 | 0.03595753 | HYPER |
| cg00353953 | chr12 | 6799541 | S_Shore | TSS1500 | ZNF384 | 7.79E-06 | 0.03747449 | HYPER |
| cg03809033 | chr2 | 216240627 | OpenSea | Body | FN1 | 7.88E-06 | 0.03765625 | hypo |
| cg11620456 | chr14 | 64062569 | S_Shore |  |  | 7.92E-06 | 0.03765625 | hypo |
| cg26888630 | chr16 | 842039 | Island | Body | CHTF18 | 8.18E-06 | 0.03860384 | hypo |
| cg02243479 | chr17 | 78859959 | N_Shelf | Body | RPTOR | 8.26E-06 | 0.03860384 | hypo |
| cg19765196 | chr3 | 105299405 | OpenSea |  |  | 8.26E-06 | 0.03860384 | hypo |
| cg04624564 | chrX | 73325368 | Island | Body | NCRNA00182 | 8.65E-06 | 0.04017231 | hypo |
| cg16577002 | chr16 | 4588414 | Island | 5'UTR | C16orf5 | 8.92E-06 | 0.04038981 | HYPER |
| cg15783848 | chr7 | 2100357 | Island | Body | MAD1L1 | 8.89E-06 | 0.04038981 | hypo |
| cg15900159 | chr13 | 24421418 | OpenSea | Body | MIPEP | 9.14E-06 | 0.04038981 | hypo |
| cg07494499 | chr17 | 837017 | OpenSea | Body | NXN | 9.06E-06 | 0.04038981 | HYPER |
| cg26465206 | chr10 | 13670022 | OpenSea | Body | PRPF18 | 8.92E-06 | 0.04038981 | hypo |
| cg03493899 | chr4 | 95133241 | S_Shelf | Body | SMARCAD1 | 9.10E-06 | 0.04038981 | hypo |
| cg20686203 | chr11 | 64883528 | N_Shore | 3'UTR | TM7SF2 | 9.05E-06 | 0.04038981 | hypo |
| cg16202181 | chr2 | 170870436 | OpenSea | Body | UBR3 | 8.89E-06 | 0.04038981 | hypo |
| cg11832534 | chr1 | 3563998 | N_Shelf | Body | WDR8 | 8.95E-06 | 0.04038981 | hypo |
| cg21938523 | chr19 | 16222446 | Island | TSS200 | RAB8A | 9.29E-06 | 0.04085052 | hypo |
| cg24423282 | chr11 | 47737085 | Island | TSS200 | AGBL2 | 9.38E-06 | 0.04100709 | HYPER |
| cg00893581 | chr5 | 132857400 | OpenSea | Body | FSTL4 | 9.59E-06 | 0.04172466 | HYPER |
| cg15720543 | chr14 | 26477646 | OpenSea |  |  | 1.04E-05 | 0.04508872 | HYPER |
| cg13858974 | chr9 | 86149293 | N_Shelf | Body | FRMD3 | 1.06E-05 | 0.04543584 | HYPER |
| cg01547531 | chr2 | 160627347 | OpenSea | 3'UTR | CD302 | 1.08E-05 | 0.04606288 | hypo |
| cg09973699 | chr10 | 70002872 | OpenSea |  |  | 1.08E-05 | 0.04606288 | HYPER |
| cg25805321 | chr2 | 67468028 | OpenSea | Body | LOC101927661 | 1.11E-05 | 0.04689962 | hypo |
| cg09830308 | chr16 | 74734321 | Island | 5'UTR | MLKL | 1.11E-05 | 0.04689962 | HYPER |
| cg27128489 | chr16 | 46865867 | S_Shore | TSS1500 | C16orf87 | 1.13E-05 | 0.04711365 | hypo |
| cg08777079 | chr18 | 20097443 | OpenSea |  |  | 1.13E-05 | 0.04711365 | hypo |
| cg06170269 | chr18 | 40976094 | OpenSea |  |  | 1.14E-05 | 0.04735689 | HYPER |
| ch.8.2343166R | chr8 | 118822913 | OpenSea | Body | EXT1 | 1.17E-05 | 0.04749736 | HYPER |
| cg02578070 | chr6 | 119669305 | N_Shore | Body | MAN1A1 | 1.17E-05 | 0.04749736 | hypo |
| cg13685139 | chr3 | 69812820 | OpenSea | Body | MITF | 1.16E-05 | 0.04749736 | hypo |
| cg18871621 | chr5 | 76991172 | OpenSea | Body | TBCA | 1.15E-05 | 0.04749736 | hypo |
| cg09175843 | chr2 | 70313417 | Island | TSS1500 | PCBP1 | 1.20E-05 | 0.04844552 | HYPER |
| cg09229210 | chr3 | 98524932 | OpenSea | Body | DCBLD2 | 1.21E-05 | 0.04885891 | hypo |
| cg03967501 | chr7 | 36430222 | S_Shore | TSS1500 | KIAA0895 | 1.22E-05 | 0.04885891 | HYPER |
| cg03220751 | chr12 | 109490328 | Island | TSS200 | USP30 | 1.24E-05 | 0.04959047 | HYPER |

Region indicates where the CpG located within the relevant gene.

TSS1500 corresponds to the region 200–1500 bases upstream of the transcriptional start site (TSS). TSS200 represents the region 0–200 bases upstream of the TSS. The term 5′UTR denotes the 5′ untranslated region, situated between the TSS and the ATG start site. 1stExon abbreviates the first exon of the gene. The term "Body" refers to the region spanning from the ATG start site to the stop codon. Lastly, the term 3′UTR stands for the 3′ untranslated region, which extends from the stop codon to the poly-A tail.

**Table S2. Detailed information of the overlapped loci.**

| **CpG** | **Location** | **Relation to Island** | **Gene Name** | **Relation to Gene** | **P value** | **FDR** | **Methylation status** |
| --- | --- | --- | --- | --- | --- | --- | --- |
| cg09175843 | chr2: 70313417 | Island | *PCBP1* | TSS1500 | 2.24E-06 | 0.01863519 | HYPER |
| cg19591512 | chr3: 134514284 | Island | *EPHB1* | 5'UTR; 1stExon | 6.55E-09 | 0.00035921 | HYPER |
| cg25497529 | chr19: 18901364 | Island | *COMP* | Body | 1.86E-09 | 0.00013896 | hypo |
| cg15157241 | chr21: 44396204 | S Shore | *PKNOX1* | 5'UTR | 1.41E-10 | 2.33E-05 | hypo |
| cg19612173 | chr12: 115121567 | Island | *TBX3* | 1stExon; 5'UTR | 4.92E-07 | 0.00674368 | HYPER |
| cg23536255 | chr6: 31276105 | N Shore |  |  | 4.56E-12 | 3.75E-06 | HYPER |
| cg05183229 | chr10: 25012543 | Island | *ARHGAP21* | 1stExon; 5'UTR | 1.02E-08 | 0.00052569 | hypo |
| cg16967640 | chr1: 165205453 | Island | *LMX1A* | Body | 3.94E-10 | 4.05E-05 | hypo |
| cg16646539 | chr4: 88141915 | Island | *KLHL8* | TSS1500 | 1.44E-07 | 0.00285607 | HYPER |
| cg13463245 | chr9: 140042657 | Island | *GRIN1* | Body | 8.09E-08 | 0.00184802 | hypo |
| cg00876541 | chr19: 10515578 | Island | *MIR1181; CDC37* | TSS1500 | 1.34E-08 | 0.00061336 | HYPER |
| cg09892203 | chr17: 64961140 | Island | *CACNG4* | 1stExon | 2.47E-07 | 0.00413848 | HYPER |
| cg24641993 | chr5: 150537046 | Island | *ANXA6* | 5'UTR | 4.32E-06 | 0.02751056 | HYPER |
| cg08313132 | chrX: 15873151 | Island | *AP1S2* | TSS200 | 7.89E-06 | 0.03772298 | HYPER |
| cg08139234 | chr1: 29240631 | N Shore | *EPB41* | TSS1500; 5'UTR | 8.91E-08 | 0.00192813 | HYPER |
| cg04182363 | chr8: 81316879 | Open Sea |  |  | 4.58E-09 | 0.00026924 | hypo |
| cg18530551 | chr5: 172670879 | Island |  |  | 8.47E-11 | 1.74E-05 | hypo |
| cg25755428 | chr19: 13875111 | Island | *MRI1* | TSS1500 | 2.18E-10 | 2.98E-05 | HYPER |
| cg17301223 | chr8: 145106438 | Island | *OPLAH* | Body | 1.97E-08 | 0.00077283 | HYPER |
| cg16487794 | chr1: 64936656 | S Shore | *CACHD1* | Body | 1.31E-08 | 0.00061336 | HYPER |
| cg11497372 | chr6: 27513092 | Open Sea |  |  | 4.59E-08 | 0.00125751 | HYPER |
| cg23658477 | chr12: 22094371 | Island |  |  | 1.02E-09 | 8.35E-05 | HYPER |
| cg10413136 | chr4: 8207119 | Open Sea | *SH3TC1* | Body | 1.95E-07 | 0.00355929 | hypo |
| cg06221470 | chr7: 2353869 | Island | *SNX8* | Body | 2.78E-06 | 0.02159913 | HYPER |
| cg13464448 | chr11: 130297513 | Island | *ADAMTS8* | 1stExon | 1.05E-07 | 0.00218602 | HYPER |
| cg18675616 | chr4: 1962842 | Open Sea | *WHSC1* | Body | 2.18E-06 | 0.01846299 | hypo |
| cg22272803 | chr7: 155989369 | Open Sea |  |  | 6.53E-08 | 0.00162626 | HYPER |
| cg10153212 | chr19: 11370382 | N Shelf | *DOCK6* | Body | 1.88E-06 | 0.01720785 | HYPER |
| cg05578055 | chr8: 16859045 | Island | *FGF20* | Body | 4.20E-08 | 0.00120145 | HYPER |
| cg07627556 | chr11: 115530590 | S Shore |  |  | 3.22E-11 | 8.82E-06 | HYPER |
| cg13745593 | chr4: 135248186 | Open Sea |  |  | 1.71E-07 | 0.0032684 | hypo |
| cg08106148 | chr6: 130339617 | N Shore | *L3MBTL3* | TSS200 | 3.08E-07 | 0.0047819 | HYPER |
| cg20227471 | chr2: 25065550 | Open Sea | *ADCY3* | Body | 3.43E-10 | 4.03E-05 | hypo |
| cg05687194 | chr16: 84178061 | Island | *LRRC50; HSDL1* | TSS1500; 5'UTR | 3.62E-06 | 0.02498652 | HYPER |
| cg15578091 | chr1: 236908064 | Open Sea | *ACTN2* | Body | 3.83E-09 | 0.00026274 | hypo |
| cg24342002 | chr20: 25604715 | Island | *NANP* | TSS200 | 1.06E-07 | 0.00218602 | HYPER |
| cg15628498 | chr15: 53082902 | Island | *ONECUT1* | TSS1500 | 1.50E-08 | 0.00064468 | HYPER |
| cg06139099 | chr10: 134499505 | Open Sea | *INPP5A* | Body | 4.82E-08 | 0.00127724 | hypo |
| cg09376537 | chr17: 48638103 | Island | *CACNA1G* | TSS1500 | 5.80E-06 | 0.03178906 | HYPER |
| cg17619755 | chr6: 31760629 | N Shelf | *VARS* | Body | 3.59E-07 | 0.00518306 | HYPER |
| cg27128489 | chr16: 46865867 | S Shore | *C16orf87* | TSS1500 | 4.19E-07 | 0.00584333 | hypo |
| cg23906144 | chr17: 1945509 | Island | *OVCA2; DPH1* | 1stExon; 3'UTR | 9.07E-06 | 0.04211833 | HYPER |
| cg12664940 | chr5: 140430875 | Open Sea | *PCDHB1* | TSS200 | 4.34E-09 | 0.00026924 | hypo |
| cg14789080 | chr13: 70367962 | Open Sea | *KLHL1* | Body | 4.47E-06 | 0.02782825 | HYPER |
| cg15900159 | chr13: 24421418 | Open Sea | *MIPEP* | Body | 1.88E-06 | 0.01720785 | hypo |
| cg08357850 | chr16: 74734885 | S Shore | *MLKL* | TSS200 | 2.81E-07 | 0.00462339 | HYPER |
| cg04135540 | chr4: 42296896 | Open Sea |  |  | 7.66E-08 | 0.00180053 | HYPER |
| cg23361197 | chr12: 95461590 | Open Sea | *NR2C1* | 5'UTR | 5.46E-06 | 0.03103037 | hypo |
| cg08344447 | chr9: 112945357 | Open Sea |  |  | 1.15E-05 | 0.04897176 | HYPER |
| cg14732655 | chr18: 71186297 | Open Sea |  |  | 4.35E-06 | 0.02751056 | HYPER |
| cg00580291 | chr13: 21606284 | Open Sea | *LATS2* | Body | 6.30E-06 | 0.03320495 | hypo |
| cg00276792 | chr16: 3208796 | Island |  |  | 1.08E-05 | 0.04761012 | hypo |
| cg19250045 | chr8: 122543761 | Open Sea |  |  | 6.26E-06 | 0.03320495 | HYPER |
| cg25303774 | chr6: 121902246 | Open Sea |  |  | 4.42E-06 | 0.02775246 | hypo |
| cg06865629 | chr18: 19409164 | Open Sea | *MIR1-2; MIB1* | TSS200; Body | 4.18E-06 | 0.02725252 | hypo |
| cg25283172 | chr2: 29038696 | Open Sea | *SPDYA* | 5'UTR; TSS200 | 6.48E-06 | 0.03352683 | hypo |
| cg16540981 | chr1: 112016558 | Open Sea | *C1orf162* | 1stExon; 5'UTR | 2.39E-07 | 0.00408801 | hypo |
| cg02664390 | chr1: 179911884 | Open Sea |  |  | 3.55E-08 | 0.00108188 | hypo |
| cg00353953 | chr12: 6799541 | S Shore | *ZNF384* | TSS1500 | 3.96E-07 | 0.00562035 | HYPER |
| cg16486564 | chr16: 46513219 | Open Sea | *ANKRD26P1* | Body | 1.70E-06 | 0.01592833 | HYPER |
| cg10767216 | chr7: 130130187 | N Shore | *MESTIT1; MEST* | Body; 5'UTR; TSS1500 | 5.33E-07 | 0.00695801 | hypo |
| cg00275967 | chr13: 106716455 | Open Sea |  |  | 1.08E-06 | 0.0112367 | HYPER |
| cg02606505 | chr3: 42689703 | Open Sea | *NKTR* | 3'UTR | 1.46E-07 | 0.00285607 | hypo |
| cg04054036 | chr14: 65273407 | Open Sea | *SPTB* | Body | 7.42E-07 | 0.00872387 | HYPER |
| cg08956303 | chr11: 45967551 | Open Sea | *PHF21A* | Body | 3.50E-07 | 0.00518306 | hypo |
| cg15071899 | chr7: 156228452 | Open Sea |  |  | 3.03E-06 | 0.02266069 | HYPER |
| cg02251850 | chr17: 78851503 | S Shore | *RPTOR* | Body | 3.07E-08 | 0.00097149 | hypo |
| cg07926598 | chr7: 4175200 | Open Sea | *SDK1* | Body | 1.38E-11 | 5.69E-06 | HYPER |
| cg07202461 | chr18: 19406765 | Open Sea | *MIB1; MIR133A1* | Body; TSS1500 | 1.18E-05 | 0.04993176 | hypo |
| cg08655465 | chr11: 13894916 | Open Sea |  |  | 3.29E-06 | 0.02373573 | hypo |
| cg22343476 | chr1: 225845563 | S Shelf |  |  | 6.68E-06 | 0.03389783 | HYPER |
| cg21106962 | chr10: 62150977 | Open Sea | *ANK3* | TSS1500; Body | 7.39E-08 | 0.00178618 | hypo |
| cg06810293 | chr6: 34660294 | N Shelf | *C6orf106* | Body | 5.70E-06 | 0.03162763 | hypo |
| cg09889646 | chr2: 48692092 | Open Sea | *PPP1R21* | Body | 2.64E-06 | 0.02069767 | hypo |
| cg10808367 | chr10: 134451193 | Open Sea | *INPP5A* | Body | 5.19E-07 | 0.00695801 | hypo |
| cg10030268 | chr2: 234465509 | Open Sea | *USP40* | Body | 5.99E-08 | 0.00153999 | hypo |
| cg15836199 | chr1: 2302005 | N Shelf | *MORN1* | Body | 8.53E-07 | 0.00947975 | hypo |
| cg11385067 | chr4: 68751543 | Open Sea |  |  | 9.25E-07 | 0.00988248 | HYPER |
| cg21186771 | chr2: 73287456 | Open Sea | *SFXN5* | Body | 9.96E-06 | 0.04551897 | hypo |
| cg03511402 | chr16: 30583225 | Island | *ZNF688* | 5'UTR; Body; 1stExon | 5.73E-06 | 0.03162763 | HYPER |
| cg13858974 | chr9: 86149293 | N Shelf | *FRMD3* | Body | 3.27E-06 | 0.02373573 | HYPER |

Region indicates where the CpG located within the relevant gene.

TSS1500 corresponds to the region 200–1500 bases upstream of the transcriptional start site (TSS). TSS200 represents the region 0–200 bases upstream of the TSS. The term 5′UTR denotes the 5′ untranslated region, situated between the TSS and the ATG start site. 1stExon abbreviates the first exon of the gene. The term "Body" refers to the region spanning from the ATG start site to the stop codon. Lastly, the term 3′UTR stands for the 3′ untranslated region, which extends from the stop codon to the poly-A tail.

**Table S3. Replicated DNA methylation loci.**

|  | | | | **Methylation status** | | |
| --- | --- | --- | --- | --- | --- | --- |
| **CpG** | **Position** | **Gene** | **FDR** | **AngII** | **IGF-1** | **DCM** |
| cg06233497 | chr11: 120200253 | *TMEM136* | 0.001 | Hypo | – | Hyper |
| cg14561362 | chr4: 146461062 | *SMAD1* | 0.001 | – | Hyper | Hypo |
| cg03870044 | chr5: 125966247 | *C5orf48* | 0.018 | Hypo | – | Hyper |
| cg01175610 | chr12: 12224246 | *BCL2L14* | 0.017 | – | Hyper | Hyper |
| cg22272803 | chr7: 155989369 | *–* | 0.015 | Hyper | Hyper | Hyper |
| cg26012716 | chr11: 86748913 | *TMEM135* | 0.021 | Hyper | – | Hypo |
| cg13864354 | chr6: 136206116 | *PDE7B* | 0.020 | – | Hypo | Hyper |
| cg16967640 | chr1: 165205453 | *LMX1A* | 0.021 | Hypo | Hypo | Hypo |
| cg13118761 | chr14: 89293608 | *TTC8* | 0.019 | Hypo | – | Hyper |
| cg05252062 | chr7: 101712308 | *CUX1* | 0.019 | – | Hypo | Hyper |
| cg15438999 | chr2: 97538522 | *–* | 0.026 | Hyper | – | Hyper |
| cg24232444 | chr13: 99545448 | *DOCK9* | 0.034 | Hypo | – | Hyper |
| cg06638795 | chr2: 942719933 | *KCNG3* | 0.034 | – | Hyper | Hypo |
| cg07202461 | chr18: 19406765 | *MIB1;MIR133A1* | 0.046 | Hypo | Hypo | Hypo |

Dashes in the “Gene” column indicate that no known gene is annotated for this CpG site.

Dashes in the “AngII” or “IGF-1” column indicate non-significance.

“Hypo” indicates hypomethylated, and “Hyper” indicates hypermethylated.

FDR, false discovery rate; DCM, dilated cardiomyopathy; chr, chromosome; and CpG, cytosine-phosphate-guanine.

**Table S4. Pathway analysis of the cardiovascular diseases (CVD) and physical activity (PA) related DNA methylation loci based on the dataset form the MESA study**

| **Term** | **Count (%)** | **P-value** | **Genes** | **Fold Enrichment** |
| --- | --- | --- | --- | --- |
| **CVD** | | | | |
| Signaling pathways regulating pluripotency of stem cells | 5 (5.2) | 0.009 | JAK1, WNT4, DVL1, ID3, PIK3CD | 6.0 |
| Choline metabolism in cancer | 4 (4.2) | 0.011 | WASF2, MTOR, PIK3CD, SLC44A5 | 6.6 |
| PI3K-Akt signaling pathway | 6 (6.2) | 0.055 | GNG12, JAK1, EFNA1, MTOR, PIK3CD | 4.8 |
| Glioma | 3 (3.1) | 0.071 | SHC1, MTOR, PIK3CD | 7.7 |
| Bacterial invasion of epithelial cells | 3 (3.1) | 0.075 | SHC1, WASF2, PIK3CD | 6.5 |
| Peroxisome | 3 (3.1) | 0.083 | PRDX1, PEX10, PEX14 | 6.1 |
| Pathways in cancer | 7 (7.3) | 0.088 | CKS1B, GNG12, JAK1, WNT4, DVL1, MTOR, PIK3CD | 3.0 |
| ErbB signaling pathway | 3 (3.1) | 0.088 | SHC1, MTOR, PIK3CD | 5.8 |
| **PA** | | | | |
| PI3K-Akt signaling pathway | 16 (3.1) | 0.024 | EPHA2, GNG5, JAK1, CASP9, COL24A1, CSF1, EFNA1, EFNA3, IL6R, LPAR3, MTOR, NGF, NRAS, PIK3CD, THEM4, THBS3 | 1.9 |
| Cell adhesion molecules (CAMs) | 9 (1.7) | 0.025 | CD2, CD58, VTCN1, CLDN19, NTNG1, NEGR1, PTPRF, SDC3, VCAM1 | 2.5 |
| Rap1 signaling pathway | 11 (2.1) | 0.036 | EPHA2, RAP1GAP, CSF1, EFNA1, EFNA3, LPAR3, MAGI3, NGF, NRAS, PIK3CD, PRKCZ | 2.1 |
| Insulin resistance | 7 (1.4) | 0.052 | MTOR, PIK3CD, PRKCZ, PTPRF, RPS6KA1, SLC2A1, SLC27A3 | 2.6 |
| Insulin signaling pathway | 8 (1.6) | 0.056 | MKNK1, SHC1, MTOR, NRAS, PIK3CD, PRKCZ, PTPRF, PKLR | 2.3 |
| Thyroid hormone signaling pathway | 7 (1.4) | 0.066 | WNT4, CASP9, MTOR, NRAS, PIK3CD, SLCA1, SLC9A1 | 2.4 |
| Axon guidance | 7 (1.4) | 0.096 | EPHA2, EPHB2, EFNA1, EFNA3, NTNG1, NRAS, SEMA4A | 2.2 |
| Inositol phosphate metabolism | 5 (1.0) | 0.100 | INPP5B, PI4KB, PIK3CD, PIP5K1A, PLCH2 | 2.8 |

**Figure S1. Sample quality control report.**

**Figure S2. Visualized interpretation of conditions categorized by the three linear regression coefficients.**

**Figure S3. Pathway analysis conducted through the functional class scoring methods using both KEGG and GO databases.**

**A.**

**IGF-1 (GO)**

**D.**

**IGF-1 (KEGG)**

**C.**

**AngII (GO)**

**AngII (KEGG)**

**B.**

**Figure S4. Validation of gene expressions for the thee mutual genes in both the current study and the cohort study.**

**C.**

**B.**

**A.**

**Figure S5. Global DNA methylation distribution and gene expressions of some selected genes in PI3K-Akt pathway.**

**Figure S6. Validation of gene expressions of some selected genes in Hippo signaling pathway.**
